# Abnormal brain cholesterol homeostasis in Alzheimer’s disease—a targeted metabolomic and transcriptomic study

**DOI:** 10.1038/s41514-021-00064-9

**Published:** 2021-06-01

**Authors:** Vijay R. Varma, H. Büşra Lüleci, Anup M. Oommen, Sudhir Varma, Chad T. Blackshear, Michael E. Griswold, Yang An, Jackson A. Roberts, Richard O’Brien, Olga Pletnikova, Juan C. Troncoso, David A. Bennett, Tunahan Çakır, Cristina Legido-Quigley, Madhav Thambisetty

**Affiliations:** 1 Clinical and Translational Neuroscience Section, Laboratory of Behavioral Neuroscience, National Institute on Aging (NIA), National Institutes of Health (NIH), Baltimore, MD USA; 2 Department of Bioengineering, Gebze Technical University, Kocaeli, Turkey; 3 Glycoscience Group, NCBES National Centre for Biomedical Engineering Science, National University of Ireland Galway, Galway, Ireland; 4 HiThru Analytics, Laurel, MD USA; 5 University of Mississippi Medical Center, Jackson, MS USA; 6 Laboratory of Behavioral Neuroscience, National Institute on Aging (NIA), National Institutes of Health (NIH), Baltimore, MD USA; 7 Department of Neurology, Duke University School of Medicine, Durham, NC USA; 8 Department of Pathology, Johns Hopkins University School of Medicine, Baltimore, MD USA; 9 Rush Alzheimer Disease Center, Rush University, Chicago, IL USA; 10 Kings College London, London, UK

**Keywords:** Alzheimer's disease, Alzheimer's disease

## Abstract

The role of brain cholesterol metabolism in Alzheimer’s disease (AD) remains unclear. Peripheral and brain cholesterol levels are largely independent due to the impermeability of the blood brain barrier (BBB), highlighting the importance of studying the role of brain cholesterol homeostasis in AD. We first tested whether metabolite markers of brain cholesterol biosynthesis and catabolism were altered in AD and associated with AD pathology using linear mixed-effects models in two brain autopsy samples from the Baltimore Longitudinal Study of Aging (BLSA) and the Religious Orders Study (ROS). We next tested whether genetic regulators of brain cholesterol biosynthesis and catabolism were altered in AD using the ANOVA test in publicly available brain tissue transcriptomic datasets. Finally, using regional brain transcriptomic data, we performed genome-scale metabolic network modeling to assess alterations in cholesterol biosynthesis and catabolism reactions in AD. We show that AD is associated with pervasive abnormalities in cholesterol biosynthesis and catabolism. Using transcriptomic data from Parkinson’s disease (PD) brain tissue samples, we found that gene expression alterations identified in AD were not observed in PD, suggesting that these changes may be specific to AD. Our results suggest that reduced de novo cholesterol biosynthesis may occur in response to impaired enzymatic cholesterol catabolism and efflux to maintain brain cholesterol levels in AD. This is accompanied by the accumulation of nonenzymatically generated cytotoxic oxysterols. Our results set the stage for experimental studies to address whether abnormalities in cholesterol metabolism are plausible therapeutic targets in AD.

## Introduction

While several epidemiological studies suggest that midlife hypercholesterolemia is associated with an increased risk of Alzheimer’s disease (AD), the role of brain cholesterol metabolism in AD remains unclear. The impermeability of cholesterol to the blood brain barrier (BBB) ensures that brain concentrations of cholesterol are largely independent of peripheral tissues^1^. This further highlights the importance of studying the role of brain cholesterol homeostasis in AD pathogenesis.

Prior epidemiologic work examining the relationship between hypercholesterolemia^1–3^ and statin use^3–5^ in AD have suggested that cholesterol metabolism may have an impact on amyloid-β aggregation and neurotoxicity as well as tau pathology^6,7^. Other studies have addressed the molecular mechanisms underlying the relationship between brain cholesterol metabolism and AD pathogenesis^8^. These studies have generally implicated oxysterols, the main breakdown product of cholesterol catabolism, as plausible mediators of this relationship^1,9^. Few studies have however tested the role of both brain cholesterol biosynthesis and catabolism in AD across multiple aging cohorts. A comprehensive understanding of cholesterol metabolism may uncover therapeutic targets as suggested by emerging evidence that modulation of brain cholesterol levels may be a promising drug target^10^.

In this study, we utilized targeted and quantitative metabolomics to measure brain tissue concentrations of both biosynthetic precursors of cholesterol as well as oxysterols, which represent BBB-permeable products of cholesterol catabolism, in samples from participants in two well-characterized cohorts—the Baltimore Longitudinal Study of Aging (BLSA) and the Religious Orders Study (ROS). We additionally utilized publicly available transcriptomic datasets in AD and control (CN) brain tissue samples to study differences in regional expression of genes regulating reactions within de novo cholesterol biosynthesis and catabolism pathways. Finally, we mapped regional brain transcriptome data on genome-scale metabolic networks to compare flux activity of reactions representing de novo cholesterol biosynthesis and catabolism between AD and CN samples.

We addressed the following key questions in this study:

1. Are brain metabolite markers of cholesterol biosynthesis and catabolism altered in AD and associated with severity of AD pathology in two demographically distinct cohorts of older individuals?
2. Are the genetic regulators of cholesterol biosynthesis and catabolism altered in brain regions vulnerable to AD pathology and are these alterations specific to AD or represent non-specific characteristics related to neurodegeneration in other diseases such as Parkinson’s disease (PD)?
3. Are predicted metabolic flux activity through reactions within cholesterol biosynthesis and catabolism altered in brain regions vulnerable to AD pathology and are these alterations specific to AD?

## Results

### Demographics

Table 1 summarizes the demographic characteristics of the BLSA and ROS samples. In the BLSA sample, the three groups—AD, cognitively normal (CN), and asymptomatic AD (ASY)—did not differ significantly in age at death, sex, *APOE* ε4 carrier status, statin use, and postmortem interval (PMI). AD samples were more likely White (race) compared to CN samples. The three groups varied significantly in the severity of neuritic plaques (CERAD scores) with the AD group showing the highest pathology, ASY intermediate, and CN with the lowest levels of pathology. CN, as expected, differed from AD in the severity of neurofibrillary tangles (Braak scores), with AD group showing the highest and CN the lowest levels of pathology.

**Table 1:** Demographic characteristics of study samples.

Baltimore Longitudinal Study of Aging (BLSA): study sample				
	Total sample, *N* = 29	CN, *N* = 8	ASY, *N* = 6	AD, *N* = 15
Age at death, mean (SD)	86.83 (9.88)	83.97 (15.06)	83.78 (6.71)	89.58 (6.99)
Age of onset, mean (SD)	–	–	–	80.88 (7.83)^a^
Disease duration, mean (SD)	–	–	–	8.70 (3.89)^a^
Sex, *n* (% female)	14 (48.28)^a^	4 (50.00)	3 (50.00)	7 (46.67)^a^
Race, *n* (% white)	27 (93.10)^a^	6 (75.00)^a,b^	6 (100.00)	15 (100.00)^b^
*APOE* e4 carrier, *n* (%)	8 (30.77)	2 (25.00)	1 (20.00)	5 (38.46)
Statin use, *n* (%)	6 (20.69)^a^	3 (37.50)	1 (16.67)	2 (13.33)^a^
CERAD, mean (SD)	1.90 (1.14)	0.25 (0.46)^b,d^	2.17 (0.41)^c,d^	2.67 (0.49)^b,c^
Braak, mean (SD)	4.00 (1.65)	2.63 (1.30)^b^	3.50 (1.64)	4.94 (1.22)^b^
Postmortem interval (hours), mean (SD)	15.48 (12.20)^a^	11.38 (6.41)	13.58 (4.36)	18.64 (16.03)^a^

Religious Orders Study (ROS): study sample				
	Total sample, *N* = 71	CN, *N* = 22	ASY, *N* = 18	AD, *N* = 31
Age at death, mean (SD)	89.52 (7.02)	87.44 (6.75)^b^	86.42 (7.65)^c^	92.81 (5.45)^b,c^
Age of onset, mean (SD)	–	–	–	89.13 (5.71)^a^
Disease duration, mean (SD)	–	–	–	3.67 (2.94)^a^
Sex, *n* (% female)	55 (77.46)^a^	13 (59.09)^b^	15 (83.33)	27 (87.10)^a,b^
Race, *n* (% white)	71 (100.00)^a^	22 (100.00)^a^	18 (100.00)	31 (100.00)
*APOE* e4 carrier, *n* (%)	19 (27.54)	4 (18.18)	6 (33.33)	9 (31.03)
Statin use, *n* (%)	41 (57.75)^a^	13 (59.09)	10 (55.56)	18 (58.06)^a^
CERAD, mean (SD)	1.82 (1.15)	0.32 (0.65)^b,d^	2.33 (0.49)^d^	2.58 (0.50)^b^
Braak, mean (SD)	3.85 (1.14)	2.91 (1.11)^b,d^	3.61 (0.85)^c,d^	4.65 (0.66)^b,c^
Postmortem interval (hours), mean (SD)	9.48 (6.14)^a^	9.59 (5.35)	10.80 (8.24)	8.67 (5.36)^a^

*AD* Alzheimer’s disease, *CN* cognitively normal, *ASY* asymptomatic AD, Disease duration: age death—age onset.

^a^*P* < 0.05 comparing BLSA to ROS (e.g., AD in BLSA compared to AD in ROS).

^b^*P* < 0.05 comparing AD to CN.

^c^*P* < 0.05 comparing AD to ASY.

^d^*P* < 0.05 comparing ASY to CN.

In the ROS sample, the three groups did not vary significantly in race, *APOE* ε4 carrier status, statin use, and PMI. Persons with AD were significantly older at death compared to both ASY and CN samples and were more likely female (sex) compared to CN. CN, as expected, differed from ASY and AD in the severity of neuritic plaque pathology (CERAD scores) and neurofibrillary tangle pathology (Braak scores) with the AD group showing the highest pathology, ASY being intermediate and CN with the lowest severity of each pathology.

Table 1 additionally summarizes differences across cohorts. Considering the total sample, BLSA and ROS varied significantly in sex, race, statin use, and PMI. Comparing by group (e.g., BLSA AD/ASY/CN compared to ROS AD/ASY/CN, respectively), BLSA and ROS samples did not vary in the age at death, *APOE* ε4 carrier status, CERAD scores, or Braak scores. BLSA AD samples compared to ROS AD samples were significantly younger at age of onset, had a longer disease duration, lower percentage females, less likely to use statins, and had a longer PMI. BLSA ASY samples did not vary from ROS ASY samples. BLSA CN samples were significantly lower percentage White (race).

### De novo cholesterol biosynthesis

In pooled primary analyses (i.e., BLSA and ROS samples combined) (Table 2), we observed significantly lower lanosterol concentration in the AD group in the MFG (AD < ASY < CN; *P* < 0.001). We additionally observed that lower lanosterol concentration in the MFG was significantly associated with higher neuritic plaque burden (*P* = 0.012) and higher neurofibrillary tangle pathology (*P* < 0.001). Brain tissue concentration of free cholesterol was not associated with disease status, neuritic plaque burden (CERAD score), or neurofibrillary tangle pathology (Braak score).

**Table 2:** Pooled associations between metabolite levels and disease status/severity of AD pathology.

	Disease group				CERAD				Braak			
	ITG		MFG		ITG		MFG		ITG		MFG	
Metabolite name	β	*P* value	β	*P* value	β	*P* value	β	*P* value	β	*P* value	β	*P* value
*De novo cholesterol biosynthesis*												
Cholesterol	.	.	−0.062	0.170	.	.	−0.037	0.292	.	.	0.007	0.806
Lanosterol	.	.	**−0.278**	**<0.001**	.	.	**−0.139**	**0.012**	−0.090	0.050	**−0.180**	**<0.001**
24,25-dihydrolanosterol	.	.	.	.	.	.	.	.	−0.032	0.333	.	.
7-dehydrocholesterol	.	.	.	.	.	.	.	.	0.160	0.147	.	.
Desmosterol												
*Cholesterol catabolism (enzymatic)*												
27-hydroxycholesterol	0.210	0.170	.	.	0.066	0.555	.	.	0.051	0.641	−0.120	0.223
4β-hydroxycholesterol	0.119	0.234	.	.	0.030	0.688	.	.	.	.	.	.
24S-hydroxycholesterol	**−0.113**	**0.011**	.	.	**−0.074**	**0.008**	.	.	**−0.094**	**<0.001**	**−0.087**	**0.042**
7α-hydroxycholesterol	0.154	0.068	.	.	**0.135**	**0.023**	.	.	−0.002	0.977	−0.109	0.066
*Cholesterol catabolism (non-enzymatic)*												
5α,6α-epoxycholesterol	**0.199**	**0.040**	0.115	0.139	0.118	0.116	0.038	0.508	.	.	.	.
5α,6β-dihydroxycholestanol	**0.213**	**0.017**	−0.155	0.088	0.105	0.096	−0.111	0.101	.	.	−0.106	0.062
5β,6β-epoxycholesterol	**0.196**	**0.013**	0.071	0.327	0.112	0.069	.	.	0.085	0.152	.	.
7-ketocholesterol	**0.304**	**0.001**	.	.	**0.179**	**0.008**	.	.	0.102	0.155	−0.035	0.537
7β-hydroxycholesterol	**0.237**	**0.005**	.	.	**0.157**	**0.010**	.	.	0.050	0.448	−0.089	0.284

*ITG* Inferior Temporal Gyrus, *MFG* Middle Frontal Gyrus; *P* < 0.05 in bold.

Negative coefficients indicate that lower metabolite concentration is significantly associated with AD, higher neuritic plaque burden (CERAD score), or higher neurofibrillary tangle pathology (Braak score). Positive coefficients indicate that higher metabolite concentration is significantly associated with AD, higher neuritic plaque burden (CERAD score), or higher neurofibrillary tangle pathology (Braak score). Blank cells indicate that results were not pooled; these are included in cohort-specific secondary analyses in Supplementary Table 1. Significant associations (*P* < 0.05) are indicated in bold. Baltimore Longitudinal Study on Aging (BLSA) sample size: AD (*n* = 15), CN (*n* = 8); Religious Orders Study (ROS) sample size: AD (*n* = 31), CN (*n* = 22); note: samples pooled in analyses.

In cohort-specific secondary analyses (Supplementary Table 1), there were no significant associations (FDR-adjusted *P* < 0.05) between any other metabolites related to de novo cholesterol biosynthesis and either disease status or severity of AD pathology.

### Cholesterol catabolism (enzymatic)

In pooled primary analyses (i.e., BLSA and ROS samples combined) (Table 2), we observed significantly lower 24S-hydroxycholesterol concentration in the AD group in the ITG (AD < ASY < CN; *P* = 0.011). We additionally observed that lower 24S-hydroxycholesterol in the ITG was significantly associated with higher neuritic plaque burden (*P* = 0.006) and lower 24S-hydroxycholesterol concentration in both the ITG (*P* = 0.002) and MFG (*P* = 0.042) were associated with higher neurofibrillary pathology. Higher 7α-hydroxycholesterol concentration in the ITG was significantly associated with higher neuritic plaque burden (*P* = 0.027).

In cohort-specific secondary analyses (Supplementary Table 1), we observed significantly lower 24S-hydoxycholesterol and 7α-hydroxycholesterol concentration in the AD group in the MFG in ROS (AD < ASY < CN; FDR-adjusted *P* = 0.036 and 0.048, respectively). Lower 7α-hydroxycholesterol concentration in the MFG in ROS was significantly associated with higher neuritic plaque burden (FDR-adjusted *P* = 0.037). Lower 4β-hydroxycholesterol concentration in the MFG in ROS was also significantly associated with higher neuritic plaque burden (FDR-adjusted *P* = 0.029).

### Cholesterol catabolism (non-enzymatic)

In pooled primary analyses (i.e., BLSA and ROS samples combined) (Table 2), we observed significantly higher 5α,6α-epoxycholesterol, 5α,6β-dihydroxycholestanol, 5β,6β-epoxycholesterol, 7-ketocholesterol, and 7β-hydroxycholesterol concentrations in the AD group in the ITG (AD > ASY > CN; *P* = 0.040, *P* = 0.017, *P* = 0.013, *P* = 0.001, *P* = 0.005 respectively). Higher 7-ketocholesterol and 7β-hydroxycholesterol concentrations in the ITG were also associated with higher neuritic plaque burden (*P* = 0.008, *P* = 0.010, respectively).

In cohort-specific secondary analyses (Supplementary Table 1), we observed significantly lower 5α,6β-dihydroxycholestanol concentration and 7β-hydroxycholesterol concentration in the AD group in the MFG in ROS (AD < ASY < CN; FDR-adjusted *P* = 0.039 and *P* = 0.039, respectively).

### Regional brain expression of genes regulating de novo cholesterol biosynthesis, cholesterol catabolism (enzymatic), and cholesterol esterification

**Fig. 1:**
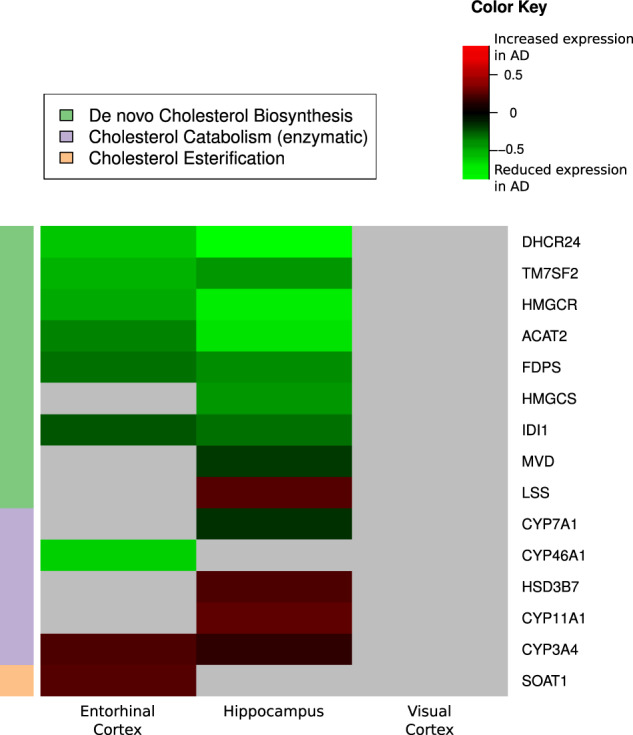
Differential brain gene expression in AD. AD Alzheimer’s disease, CN control, ERC entorhinal cortex. Differential brain gene expression of de novo cholesterol biosynthesis, catabolism (enzymatic), and esterification in AD. Summary of genes differentially expressed in selected brain regions in AD compared to CN across three pathways (de novo cholesterol biosynthesis, cholesterol catabolism (enzymatic), and cholesterol esterification). Green shading indicates that gene expression was significantly reduced in AD compared to CN. Red shading indicates that gene expression was significantly increased in AD compared to CN. Gray shading indicates gene expression was not significantly different between AD and CN. Gene Expression Ominbus (GEO) data sample size: ERC: AD (*n* = 25), CN (*n* = 52); hippocampus: AD (*n* = 29), CN (*n* = 56); visual cortex: AD (*n* = 18), CN (*n* = 12).

As indicated in the Fig. 1 heatmap, for de novo cholesterol biosynthesis we observed significantly altered gene expression (FDR-adjusted *P* value <0.05) in AD in 9 out of 20 genes. The majority of significantly altered genes (eight out of nine) showed lower gene expression in AD relative to CN (AD < CN) in the ERC and/or hippocampus. We observed no significant associations in the control region (i.e., visual cortex).

For cholesterol catabolism (enzymatic), we observed significantly altered gene expression (FDR-adjusted *P* value <0.05) in five out of ten genes. Three had higher gene expression in AD compared to CN (AD > CN) and two had lower gene expression in AD relative to CN (AD < CN) in the ERC and/or hippocampus. We observed no significant associations in the control region (i.e., visual cortex).

For cholesterol esterification, we observed significantly altered gene expression in one out of one gene with higher gene expression in AD compared to CN (AD > CN) in the ERC. We observed no significant associations in the control region (i.e., visual cortex). Significant genes are included in Fig. 1 and log-fold changes and *P* values for all genes are included in Supplementary Table 2.

Of the 15 genes that were significantly altered in AD, we did not observe significantly altered expression (FDR-adjusted *P* value <0.05) in PD compared to CN in the substantia nigra (Supplementary Table 3).

### Genome-scale metabolic network modeling of reactions within de novo cholesterol biosynthesis, catabolism (enzymatic), and esterification

In Table 3, we summarize results of genome-scale metabolic network modeling of reactions within de novo cholesterol biosynthesis, catabolism (enzymatic), and esterification. Out of 177 reactions catalyzed by the 31 a priori specified genes, 16 were significantly (*P* < 0.05) altered in AD in the ERC and/or hippocampus including 15 within the de novo cholesterol biosynthesis pathway (3 in pre-squalene and 12 in post-squalene) and 1 within the cholesterol catabolism (enzymatic) pathway. The majority of reactions within the de novo cholesterol biosynthesis pathway (14/15) were decreased in AD compared to CN. Within the cholesterol catabolism (enzymatic) pathway, 1/1 was increased in the AD hippocampus. The majority of reactions within the control region—visual cortex—(15/16) were not significantly different between AD and CN. Reactions related to cholesterol esterification were not predicted to be significantly altered between the two groups in any of the brain regions examined. Supplementary Table 4 includes iMAT-based metabolic network modeling results from all 177 reactions in AD and CN samples.

**Table 3:** iMAT-based metabolic network modeling of cholesterol synthesis and catabolism in AD.

			ERC		Hippocampus		Visual cortex	
Gene	Human GEM rxn ID	GEM reaction	Odds ratio	*P* value	Odds ratio	*P* value	Odds ratio	*P* value
*De novo cholesterol biosynthesis (pre-squalene mevalonate pathway)*								
ACAT2	HMR_1434	acetoacetyl-CoA[c] + CoA[c] <=> 2 acetyl-CoA[c]	1.500	1.000	**0.070**	**0.006**	0.000	0.510
HMGCS1	HMR_1437	acetoacetyl-CoA[c] + acetyl-CoA[c] + H2O[c] => CoA[c] + H^+^[c] + HMG-CoA[c]	0.721	0.603	**0.178**	**0.028**	1.018	1.000
HMGCR	HMR_1440	2 H^+^[c] + HMG-CoA[c] + 2 NADPH[c] => (R)-mevalonate[c] + CoA[c] + 2 NADP^+^[c]	0.522	0.322	**0.087**	**0.016**	0.000	0.265
*De novo cholesterol biosynthesis (post-squalene mevalonate pathway including Bloch and Kandutch-Russell)*								
SC5D	7DHCHSTEROLtr	7-dehydrocholesterol[r] <=> 7-dehydrocholesterol[c]	**0.353**	**0.049**	0.518	0.204	1.786	0.676
DHCR7	HMR_1565	H^+^[c] + NADPH[c] + 7-dehydrocholesterol[c] => cholesterol[c] + NADP^+^[c]	**0.248**	**0.009**	0.308	0.097	3.929	0.363
DHCR7	DHCR72r	H^+^[r] + NADPH[r] + 7-dehydrocholesterol[r] => cholesterol[r] + NADP^+^[r]	**0.308**	**0.023**	**0.171**	**0.008**	1.786	0.676
SC4MOL, SC5D	C14STRr	H^+^[r] + NADPH[r] + 4,4-dimethyl-5alpha-cholesta-8,14,24-trien-3-beta-ol[r] => NADP^+^[r] + 14-demethyllanosterol[r]	**0.144**	**0.001**	**0.121**	**0.000**	**0.134**	**0.021**
SC4MOL	C4STMO2Pr	NADP^+^[r] + O2[r] + 3-keto-4-methylzymosterol[r] => CO2[r] + H^+^[r] + NADPH[r] + zymosterol Intermediate 2[r]	0.750	1.000	**0.000**	**0.047**	NA	1.000
SC5D	HMR_1516	5alpha-cholesta-7,24-dien-3-beta-ol[c] + H^+^[c] + NADPH[c] + O2[c] => 7-dehydrodesmosterol[c] + 2 H2O[c] + NADP^+^[c]	**0.188**	**0.002**	**0.186**	**0.006**	Inf	0.265
SC5D	LSTO1r	H^+^ [r] + NADPH[r] + O2[r] + 5alpha-cholesta-7,24-dien-3-beta-ol[r] => 2 H2O[r] + NADP^+^[r] + 7-dehydrodesmosterol[r]	**0.000**	**0.031**	NA	1.000	0.000	0.510
DHCR7	HMR_1519	7-dehydrodesmosterol[c] + H^+^[c] + NADPH[c] => desmosterol[c] + NADP^+^[c]	**0.188**	**0.002**	**0.186**	**0.006**	Inf	0.265
DHCR7	DHCR71r	H^+^[r] + NADPH[r] + 7-dehydrodesmosterol[r] => NADP^+^ [r] + desmosterol[r]	**0.000**	**0.031**	NA	1.000	0.000	0.510
DHCR24	HMR_1526	desmosterol[c] + H^+^[c] + NADPH[c] => cholesterol[c] + NADP^+^[c]	**0.103**	**0.036**	0.000	0.341	0.000	0.510
DHCR24	DSREDUCr	H^+^[r] + NADPH[r] + desmosterol[r] => cholesterol[r] + NADP^+^[r]	**0.103**	**0.036**	0.000	0.341	0.000	0.510
DHCR24	DSMSTEROLtr	desmosterol[r] => desmosterol[c]	1.083	1.000	**2.824**	**0.035**	0.000	0.139
*Cholesterol catabolism (enzymatic)*								
HSD3B7	HMR_1738	cholest-5-ene-3-beta,7alpha,24(S)-triol[c] + NAD^+^[c] => 4-cholesten-7alpha,24(S)-diol-3-one[c] + H^+^[c] + NADH[c]	**4.164**	**0.021**	2.724	0.116	2.400	0.288

*GEM* genome-scale metabolic model, *Human-GEM rxn ID* Human GEM reaction ID is searchable in metabolicatlas.org and indicates the specific reaction equation and additional reaction details, *AD* Alzheimer’s disease, *CN* control, *ERC* entorhinal cortex, *[c]* cytoplasm, *[m]* mitochondria, *[r]* endoplasmic reticulum; *P* < 0.05 in bold.

Significant (*P* < 0.05) odds ratios <1.0 indicate that the reaction is less active in AD compared to CN; significant odds ratios >1.0 indicate that the reaction is more active in AD compared to CN. Note that all significant de novo cholesterol biosynthesis reactions other than DSMSTEROLtr are less active in AD compared to CN. “Inf” indicates that one of the values for calculating the odds ratio (either the number of AD or CN samples that were either active or inactive for a particular reaction) was 0. “NA” indicates two of the values for calculating the odds ratio (the number of AD and CN samples that were either active or inactive for a particular reaction) was 0. Significant associations (*P* < 0.05) are indicated in bold. Gene Expression Ominbus (GEO) data sample size: ERC: AD (*n* = 25), CN (*n* = 52); hippocampus: AD (*n* = 29), CN (*n* = 56); visual cortex: AD (*n* = 18), CN (*n* = 12).

Genome-scale metabolic network modeling in PD samples relative to CN (in the substantia nigra) of the 16 reactions that were significantly altered in AD did not reveal any significantly altered reactions (Supplementary Table 5).

**Fig. 2:**
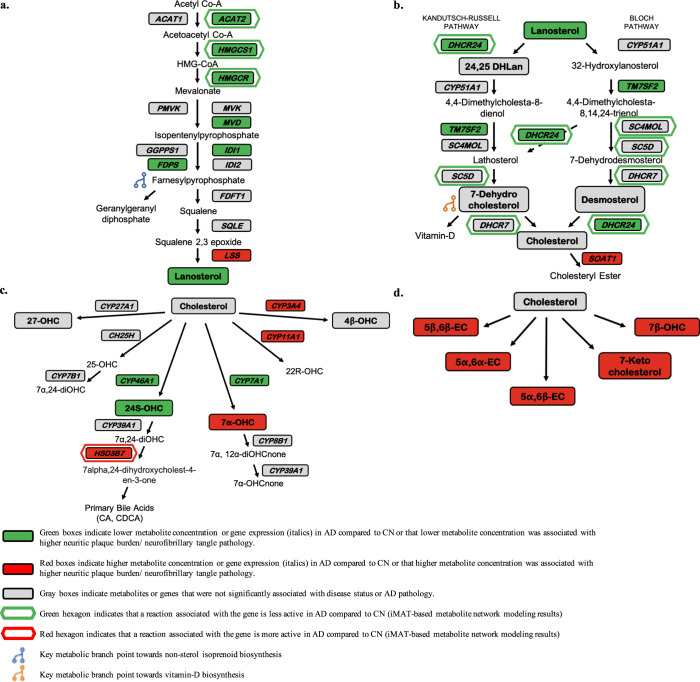
Alterations in brain metabolite concentrations and brain gene expression in AD. Alterations in brain metabolite concentrations and brain gene expression related to cholesterol biosynthesis and catabolism in AD. Metabolites indicated in bold (non-italics) and in a box (e.g., Lanosterol) were measured and detectable in the study in the ITG and MFG in the BLSA and ROS cohorts. Genes indicated in bold and in a box (e.g., CYP46A1) were measured and detectable in the ERC, hippocampus, and visual cortex in GEO datasets. Metabolites or genes not in bold and not in a box (e.g., Lathosterol) were not measured or detectable. Genes indicated in a hexagon (e.g., HSD3B7) regulate reactions that are predicted by metabolic network modeling to be significantly different between AD and CN samples. **a** De novo cholesterol biosynthesis (pre-squalene mevalonate pathway). Acetyl CoA acetyl-coenzyme A, ACAT1 acetyl-coenzyme A acetyltransferase 1, ACAT2 acetyl-coenzyme A acetyltransferase 2, acetoacetyl CoA acetoacetyl-coenzyme A, HMGCS1 3-hydroxy-3-methylglutaryl-coenzyme A synthase 1, HMG-CoA 3-hydroxy-3-methylglutaryl-coenzyme A, HMGCR 3-hydroxy-3-methylglutaryl-coenzyme A reductase, PMVK phosphomevalonate kinase, MVK mevalonate kinase, GGPPS1 geranylgeranyl diphosphate synthase 1, IDI1 isopentenyl-diphosphate delta isomerase 1, FDPS farnesyl-diphosphate synthase, IDI2 isopentenyl-diphosphate delta isomerase 2, FDFT1 farnesyl-diphosphate farnesyltransferase 1, SQLE squalene epoxidase, LSS lanosterol synthase. **b** De novo cholesterol biosynthesis (post-squalene mevalonate pathway, including the Bloch and Kandutsch–Russell pathways) and cholesterol esterification. DHCR24 24-dehydrocholesterol reductase, CYP51A1 cytochrome P450 family 51 subfamily A member 1, 24,25 DHLan 24,25-dihydrolanosterol, TM7SF2 transmembrane 7 superfamily member 2, SC4MOL methylsterol monooxygenase 1, SC5D sterol-C5-desaturase, DHCR7 7-dehydrocholesterol reductase, SOAT1 sterol O-acyltransferase 1. **c** Cholesterol catabolism (enzymatic). CYP27A1 cytochrome P450 family 27 subfamily A member 1, CYP3A4 cytochrome P450 family 3 subfamily A member 4, 4β-OHC 4β-hydroxycholesterol, 27-OHC 27-hydroxycholesterol, CH25H cholesterol 25-hydroxylase, CYP11A1 cytochrome P450 family 11 subfamily A member 1, 22R-OHC 22R-hydroxycholesterol, 25-OHC 25-hydroxycholesterol, CYP7B1 cytochrome P450 family 7 subfamily B member 1, 7α, 24-diOHC 7α, 24-dihydroxycholesterol, CYP46A1 cytochrome P450 family 46 subfamily A member 1, CYP7A1 cytochrome P450 family 7 subfamily A member 1, 24S-OHC 24S-hydroxycholesterol, CYP39A1 cytochrome P450, family 39, subfamily A member 1, 7a-OHC 7α-hydroxycholesterol, CYP8B1 cytochrome P450, family 8, subfamily B, member 1, 7α,12α-diOHCnone 7α,12α-dihydroxycholestenone, HSD3B7 3-beta-hydroxysteroid dehydrogenase type 7, 7α-OHCnone 7α-hydroxycholestenone, CA cholic acid, CDCA chenodeoxycholic acid. **d** Cholesterol catabolism (non-enzymatic). 7β-OHC 7β-hydroxycholesterol, 5α,6α-EC 5α,6α-epoxycholesterol, 5β,6β-EC 5β,6β epoxycholesterol, 5α,6β-EC 5α,6β epoxycholesterol.

Figure 2a–d summarizes metabolite, gene expression, and iMAT-based metabolic network modeling results in pathway-specific figures. For all metabolomic, gene expression, and metabolic flux results, significant associations where higher metabolite concentration, higher gene expression, or increased flux in a reaction are associated with AD are indicated in red. Significant associations where lower metabolite concentration, lower gene expression, or reduced flux in a reaction are associated with AD are indicated in green.

## Discussion

Despite the well-established association between hypercholesterolemia and AD risk, the role of brain cholesterol metabolism in AD pathogenesis remains unclear. Understanding the relevance of brain cholesterol homeostasis in AD may provide insights into effective disease-modifying treatments.

Our results suggest that while brain levels of free cholesterol are unchanged in AD, both de novo cholesterol biosynthesis and catabolism are impacted by the disease. Metabolite levels and gene expression associated with cholesterol biosynthesis are largely reduced in AD in brain regions vulnerable to pathology. Similarly, cholesterol breakdown through enzymatic conversion to its principal catabolic product, 24S-hydroxycholesterol is also reduced in AD. Furthermore, our metabolomic and differential gene expression results are supported by metabolic network modeling that suggests both reduced cholesterol biosynthesis as well as an increase in conversion of cholesterol to primary bile acids in AD. In addition, our results indicate increased non-enzymatic cholesterol catabolism in AD, suggesting a shift towards pathways that may generate potentially cytotoxic oxysterols as well as enhanced cholesterol esterification.

Our results are derived from metabolite data acquired across two longitudinally followed cohorts of older adults from distinct study populations differing in important demographic and biologic characteristics (i.e., race and sex) as well as exposure to statin therapy and PMI. Converging results from these two independent cohorts, therefore, suggest that our observations on dysregulation of cholesterol homeostasis likely reflect fundamental features of AD pathogenesis.

We additionally assessed whether our results were specific to AD by performing identical analyses on gene expression data in a non-AD neurodegenerative disease, by using brain tissue samples from PD patients. We found that gene expression of enzymes regulating de novo cholesterol biosynthesis and catabolism are not altered in the substantia nigra in PD suggesting that these changes may be relatively specific to brain regions vulnerable to AD pathology.

We observed significantly lower levels of the principal cholesterol precursor, lanosterol in AD as well as significant associations between lower lanosterol concentrations and greater severity of both neuritic plaque burden and neurofibrillary pathology. The de novo synthesis of cholesterol from acetyl CoA occurs through a series of enzymatic reactions in the mevalonate pathway (Fig. 2a) that first generate squalene (i.e., pre-squalene mevalonate pathway), which is subsequently converted to lanosterol (i.e., post-squalene mevalonate pathway). Lanosterol is the first steroidal intermediate metabolite in cholesterol biosynthesis and is converted to cholesterol by sequential enzymatic reactions through two distinct, but closely related pathways: the Bloch and Kandutsch–Russell pathways (Fig. 2b).

Our finding that AD is associated with reduced synthesis of lanosterol can be interpreted in the context of recent evidence that biosynthetic precursors of cholesterol may exert neuroprotective roles. Lanosterol has been shown to induce mitochondrial uncoupling and autophagy, promoting neuronal survival in a mouse model of Parkinson’s disease^11^. Similarly, increasing levels of lanosterol have been shown to reduce neuronal atrophy and improve motor deficits in a mouse model of Huntington’s disease as well as the survival of striatal cells overexpressing Huntingtin protein^12^. Our finding that this cholesterol precursor is depleted in the AD brain in regions vulnerable to pathology suggests that mitochondrial function and autophagy may be disrupted leading to neurodegeneration. Interestingly, the accumulation of mutant amyloid precursor protein (APP) and Aβ in hippocampal neurons has been shown to induce impairment in both mitochondrial structure and function as well as defective mitophagy and autophagy with synaptic loss^13^.

Despite a decrease in brain tissue concentration of lanosterol, we found no significant differences in absolute concentrations of free cholesterol in AD. Our results are broadly consistent with prior studies that have measured free cholesterol in the AD brain^14–16^. Some prior reports have however observed higher brain cholesterol concentrations in AD. Xiong et al. assayed both free and esterified cholesterol levels in a small sample of AD and CN brains using enzymatic methods and reported higher concentrations in AD^17^. However, it is unclear whether these assays were performed in specific brain regions or measured total brain cholesterol concentrations. In a smaller study, Cuttler et al. reported higher concentrations of cholesterol in the MFG in AD samples relative to CN^18^.

In order to further assess whether de novo cholesterol biosynthesis is altered in AD, we tested differences in gene expression of enzymatic regulators of these reactions between AD and CN samples in the hippocampus, ERC, and visual cortex.

Broadly, we observed a significant reduction in expression of several genes catalyzing reactions in de novo cholesterol biosynthesis in the hippocampus and ERC in AD, while no alterations were detected in the visual cortex. These included genes encoding enzymes catalyzing reactions leading to the synthesis of the earliest cholesterol precursors (Fig. 2a), including acetoacetyl CoA (catalyzed by ACAT1/2—cytosolic acetyl-coenzyme A acetyltransferases), the biosynthetic precursor of hydroxymethyl-glutaryl (HMG)-CoA. We also observed a significant reduction in regional brain expression of the hydroxymethyl-glutaryl (HMG)-CoA synthase (HMGCS) gene in the hippocampus and the HMG-CoA reductase (HMGCR) gene in both the ERC and hippocampus in AD. HMGCR catalyzes the formation of mevalonate from HMG-CoA, the rate-limiting step in cholesterol biosynthesis in the endoplasmic reticulum (ER), and the target of statin drugs used to lower LDL cholesterol levels in plasma. These findings are especially relevant in the context of previous epidemiological studies that have shown associations between the rs3846662 single-nucleotide polymorphism (SNP) in HMGCR and AD risk^19,20^.

In addition to reduced de novo cholesterol biosynthesis through the pre-squalene mevalonate pathway in AD (Fig. 2a), we also observed significantly reduced gene expression of enzymes involved in the synthesis of farnesylpyrophosphate (FPP), a key precursor of non-sterol isoprenoids in the ERC and hippocampus. These include isopentenyl-diphosphate isomerase (IDI) and farnesyl-diphosphate synthase (FDPS). FPP is an important metabolic branch point at the intersection of both cholesterol and non-sterol isoprenoid biosynthesis^21^. Our findings, therefore, suggest that key biochemical reactions determining the biosynthetic fates of both cholesterol and non-sterol isoprenoids are impaired in AD. These results add to growing evidence implicating perturbations in isoprenoid metabolism in AD pathogenesis^22^. Until recently, dysregulation in isoprenoid metabolism has received relatively little attention in comparison to cholesterol metabolism in the pathogenesis of AD. The isoprenoids, FPP, and geranylgeranyl pyrophosphate (GGPP) participate in prenylation reactions—an important post-translational modification of several proteins including the small GTPases, which serve as molecular switches in numerous signaling pathways relevant to AD^23^. Interestingly, in a previous proteomics study performed in the same BLSA samples as in our current report, we showed reduced levels of the GTPase signaling proteins, RHOB, and G protein subunit alpha i protein (GNAI1) in the frontal cortex in AD^24^. The role of Rho GTPases as regulators of synaptic plasticity may be especially relevant in interpreting our findings in the context of AD pathogenesis^25^.

In the post-squalene cholesterol biosynthesis pathway (Fig. 2b), we additionally found lower expression of the DHCR24 gene in the hippocampus and ERC in AD. DHCR24 was originally identified by differential mRNA display as a gene whose expression is selectively reduced in AD within regions vulnerable to AD pathology and was named Selective Alzheimer’s Disease Indicator 1 (Seladin-1)^26^. Although subsequent microarray studies have reported inconsistent results on DHCR24 expression in AD, accumulating evidence suggests that DHCR24 may exert pleiotropic effects on several molecular mechanisms relevant to AD. While DHCR24 and its substrate, desmosterol play key roles in cholesterol homeostasis, DHCR24 also has reactive oxygen species (ROS)-scavenging activity and may protect against Aβ-induced neurotoxicity and apoptosis by inhibiting caspase-3 activation^27^. Reduced DHCR24 gene expression in regions vulnerable to AD pathology may therefore indicate greater susceptibility to ROS, Aβ-induced neurotoxicity, apoptosis, and neurodegeneration.

As brain cholesterol homeostasis likely reflects net effects of both cholesterol biosynthesis and catabolism, we were also interested in assessing concentrations of metabolite markers of cholesterol breakdown. We found that cholesterol breakdown through enzymatic conversion to its principal catabolic product, 24S-hydroxycholesterol (Fig. 2c) is lower in AD, and lower 24S-hydroxycholesterol concentration is also associated with greater severity of both neuritic plaque and neurofibrillary pathology. The conversion of cholesterol to 24S-hydroxycholesterol is catalyzed by the neuron-specific enzyme CYP46A1 and this reaction represents the primary metabolic route for elimination of cholesterol from the brain across the BBB into the peripheral circulation^28,29^. Our findings are consistent with accumulating evidence that 24S-hydroxycholesterol may play important roles as a modulator of Aβ production, tau phosphorylation and neuronal death as well as cognitive performance^30,31^.

We also observed alterations in brain tissue concentrations of nonenzymatically generated oxysterols in AD (Fig. 2d). These included 7α-hydroxycholesterol (which can also be generated enzymatically by CYP7A1)^32^ and 7β-hydroxycholesterol, both of which were also significantly associated with severity of neuritic plaque pathology. Other nonenzymatically generated oxysterols whose concentrations were higher in AD included 5α,6α-epoxycholesterol, 5α,6β-dihydroxycholestanol, and 5β,6β-epoxycholesterol. Our results are relevant in the context of prior studies, suggesting that these oxysterol species may mediate cytotoxicity, apoptosis, oxidative stress and chronic inflammation^32–34^. While the precise mechanisms generating cytotoxic oxysterols in the brain remain to be identified, it is interesting that both APP and Aβ have been shown to oxidize cholesterol^33^. Furthermore, Aβ:copper complexes in lipid rafts promote the catalytic oxidation of cholesterol to generate oxysterols that may trigger hyperphosphorylation of tau and accumulation of neurofibrillary tangles^35,36^.

One previous study utilized mass spectrometry-based assays of cholesterol precursors, free cholesterol, and oxysterols in the brain in AD in comparison to CN samples. In samples from the ROS study, Hascalovici et al. used gas chromatography–mass spectrometry (GC–MS) to assay these metabolites in the frontal cortex in AD, MCI, and CN samples^16^. They however did not report any significant group differences in the concentrations of cholesterol precursors, free cholesterol, or oxysterols in their study. It is likely that differences in assay methodology (GC–MS versus UHPLC–MS/MS) may account for the inconsistency between these prior findings and our current results. Testa et al.^37^ utilized isotope dilution gas chromatography/mass spectrometry to measure enzymatically and nonenzymatically generated oxysterol concentrations from the frontal and occipital cortices in AD (*N* = 13) and CN (*N* = 4) brains. They found that levels of several oxysterols were associated with disease progression. These prior findings are broadly consistent with our current report.

Our transcriptomics analyses compared gene expression levels of several enzymes regulating synthesis of oxysterols in the brain (Fig. 2c). While the expression of many of these genes was similar in the AD and CN groups, it is striking that we find lower gene expression of CYP46A1, in the ERC in AD. CYP46A1 is the neuron-specific, rate-limiting enzyme in the elimination of cholesterol^29,38^ through its conversion to 24S-hydroxycholesterol^39^ and plays a key role in regulating brain cholesterol levels. Inactivation of CYP46A1 has been shown to lower cholesterol efflux from the brain leading to a compensatory decrease in de novo cholesterol biosynthesis^40^. This compensatory reduction in cholesterol synthesis appears to be important in maintaining steady-state cholesterol levels in the brain in response to CYP46A1 inactivation. Our current results showing unaltered concentrations of free cholesterol in the brain in AD despite reduced expression of CYP46A1 may thus be mediated by a compensatory reduction in de novo cholesterol biosynthesis as suggested by reduced concentrations of lanosterol, the early biosynthetic precursor of cholesterol. Of relevance to our current findings are also previous studies that support a role for CYP46A1 beyond cholesterol turnover as 24S-hydroxycholesterol^39,41^ is a potent modulator of NMDARs which are critical for synaptic plasticity and memory^41,42^. Recent studies have suggested that CYP46A1 plays a key role in the preservation of cognitive performance during aging and may be a promising target of disease-modifying treatments for AD^43^. Female mice overexpressing CYP46A1 showed improved measures of spatial memory during aging, modulation of NMDA receptor activity, and improved markers of synaptic integrity^44^. Activation of CYP46A1 by low-dose Efavirenz, a non-nucleoside reverse transcriptase inhibitor is a therapeutic target that is currently under evaluation in a randomized clinical trial in patients with mild cognitive impairment due to AD^43,45^. An exciting finding from a recent study by van der Kant and colleagues in induced pluripotent stem cell (iPSC)-derived neurons suggests that reducing levels of cholesterol esters through activation of CYP46A1 by Efavirenz reduced both p-tau and Aβ secretion^46^. These results raise the exciting possibility that CYP46A1 activation and conversion of cholesterol to 24S-hydroxycholesterol^39^ may be a therapeutic mechanism targeting both the principal pathological processes in AD^47^.

While the predominant mechanism of cholesterol elimination from the brain is through its conversion to 24S-hydroxycholesterol^39^ by CYP46A1, a small fraction is esterified for storage through the enzymes sterol O-acyltransferase 1 (SOAT1) (also called Acyl-CoA:cholesterol acyltransferase 1; ACAT1) and lecithin:cholesterol acyltransferase (LCAT)^48,49^. It is interesting that we find increased gene expression of SOAT1 in AD samples in the ERC. Inhibition of ACAT1 has received attention as a promising therapeutic target in AD and is believed to reduce amyloidogenic processing of APP by increasing the conversion of unesterified cholesterol to 24S-hydroxycholesterol^39^ by CYP46A1^50^. Furthermore, polymorphisms in the SOAT1 gene have been previously associated with AD risk, brain amyloid load and CSF cholesterol concentrations^51^. Our finding of increased gene expression of SOAT1 in the ERC in AD suggests that it may promote the accumulation of cholesterol esters within the endoplasmic reticulum and promote amyloidogenic processing of APP.

While regional differences in brain tissue abundance of metabolite levels and differential gene expression can provide insights into metabolic dysregulation in AD, these analyses only provide a limited view of cholesterol metabolism. They do not account for interactions between cholesterol metabolism and other biochemical pathways, consider interactions between reactions within the cholesterol biosynthesis/catabolism pathways, or identify an increase or decrease in rates of associated reactions. Therefore, to develop a systems-level overview of cholesterol metabolism in AD, we mapped regional brain transcriptomic data to a genome-scale metabolic network using iMAT in order to predict the relative activity/inactivity of reactions catalyzed by specific genetic regulators of cholesterol synthesis and catabolism. These results broadly support our interpretation that lower biosynthesis of cholesterol, as well as reduced breakdown, are characteristic biochemical abnormalities in AD. They also extend these findings by suggesting that there may be increased conversion of 24S-hydroxycholesterol to primary bile acids in the AD brain through 3β-hydroxysteroid isomerase (HSD3B7). This enzyme catalyzes the inversion of the 3β-hydroxyl group of cholesterol to the 3α-hydroxyl group of bile acids and is the converging step of bile acid synthesis through the classical, alternative, and neural pathways^52,53^. These findings add to growing evidence of abnormal brain bile acid metabolism and signaling in both AD and other neurodegenerative diseases^52,54,55^. In a recent publication using brain tissue samples from the BLSA included in this study, we reported higher concentrations of the primary bile acids, cholic acid, and chenodeoxycholic acid in AD samples relative to CN^56^. In addition, a study using similar iMAT-based analysis identified significant differences between AD and CN in bile acid-associated reactions using transcriptomic data across multiple cohorts and identified HSD3B7 as significantly altered in AD^57^. Together, these results point to a shift in cholesterol catabolism toward the enhanced synthesis of bile acids in AD and an accompanying reduction in 24S-hydroxycholesterol levels that may compromise synaptic plasticity and accelerate cognitive impairment through perturbation of NMDA receptor activity^41^.

Using targeted and quantitative metabolomics assays of brain tissue samples from two well-characterized longitudinal cohorts in combination with regional brain gene expression and metabolic network modeling, we show that AD is associated with pervasive abnormalities in cholesterol biosynthesis and catabolism. Our findings suggest a disease model where reduced de novo cholesterol biosynthesis may occur in response to impaired enzymatic cholesterol catabolism and efflux to maintain brain cholesterol levels in AD. While reduced cholesterol biosynthesis may enhance mitochondrial dysfunction and impair autophagy, reduced cholesterol conversion to 24S-hydroxycholesterol may increase amyloidogenic processing of APP, tau phosphorylation, and neuronal death. These perturbations appear to be accompanied by the accumulation of nonenzymatically generated cytotoxic oxysterols in AD that may further exacerbate oxidative damage and neuroinflammation. This model presents testable hypotheses in experimental studies that can address whether these abnormalities in cholesterol metabolism may be caused by disease or act as primary drivers of AD pathogenesis. These follow-up experimental studies are also critical to identify cholesterol metabolism-related therapeutic targets in AD.

There are important limitations to our study. While we assayed the primary metabolites associated with cholesterol biosynthesis and breakdown, these represent only a subset of the total number of metabolites in these pathways. Therefore, our interpretation of the results is restricted to the metabolites that could be reliably detected and measured. Furthermore, as our metabolomic and gene expression analyses were cross-sectional, we were unable to directly test how AD progression may impact changes in metabolite concentrations or their genetic regulation.

Another limitation was that the analyses of gene expression were performed on publicly available datasets in brain regions distinct from the BLSA or ROS where metabolite levels were assayed. The limited availability of tissue samples from the ERC and hippocampus in BLSA and ROS precluded metabolomics assays on these regions in our study. The regions chosen for gene expression analyses for both AD and PD were however disease-specific and symptom proximate regions (ERC/hippocampus in AD; substantia nigra in PD) that may allow us to derive disease-specific insights about abnormal metabolism in AD.

Another important consideration is that prior studies have indicated that many community-dwelling older individuals have multiple brain pathologies^58^. This makes it challenging to conclusively attribute observed metabolomic changes in our study to specific pathologies. However, our use of two independent cohorts where both AD and CN samples were defined using standardized clinical and neuropathologic diagnoses, and our inclusion of PD as a non-AD neurodegenerative disease comparison provides additional support that our findings may be specific to AD and associated pathologies.

We also acknowledge that we chose to focus our attention on findings that were convergent between the BLSA and ROS cohorts as these are more likely to be related to AD-associated pathological changes. There are however divergent associations between the two cohorts that may be driven primarily by interactions between metabolic abnormalities and demographic differences such as sex and race as well as non-AD pathologies that merit further investigation.

Despite these limitations, our integrated analyses of regional brain metabolomic and transcriptomic data enhance understanding of dysregulated cholesterol metabolism in AD, provide valuable opportunities to test a priori hypotheses in experimental models in future studies, and may pave the way for the discovery of treatments targeting abnormal cholesterol metabolism in AD.

## Methods

### Participants: BLSA

The BLSA is a prospective cohort study that began at the National Institute on Aging (NIA) in 1958^59,60^.

Evaluations are scheduled every two years. As of 2003 participants over the age of 80 are scheduled for evaluations every year.

Autopsy sample characteristics have been described in detail previously^61^, including differences compared to the overall BLSA cohort^62^, neuropathologic assessment of AD pathology^63^, and clinical diagnoses at consensus case conferences^64^.

BLSA autopsy participants were classified into three groups based on the below criteria:

1) AD participants (*n* = 15) had a clinical diagnosis of either AD or MCI due to AD within 1 year of death and a CERAD pathology score of >1 (i.e., CERAD B or C).

2) Cognitively normal (CN) participants (*n* = 8) had normal cognition within 1 year of death and a CERAD pathology score < =1 (i.e., CERAD 0 or A). CN participants did not have significant amyloid pathology at autopsy and cognitive impairment during life.

3) Asymptomatic AD (ASY) participants (*n* = 6) had a clinical diagnosis of normal cognition within 1 year of death and a CERAD pathology score >1 (i.e., CERAD B or C). ASY participants had characteristic AD brain pathology (neuritic plaques and neurofibrillary tangles) and did not have cognitive impairment during life. This group has been described previously^65^. Table 1 describes the demographic characteristics of the BLSA sample.

The BLSA study protocol has ongoing approval from the Institutional Review Board of the National Institute of Environmental Health Science, National Institutes of Health. Written informed consent was obtained at each visit from all participants.

### Participants: ROS

The ROS is a longitudinal, clinical and pathological cohort study of individuals within religious communities across the US with the goal of linking AD risk factors with incident clinical and neuropathologic phenotypes^66^. The study began enrolling participants without known dementia in 1994. Study methods including recruitment have been detailed previously^67^. Follow-up and autopsy rates exceed 90% and 85%, respectively^68^.

Our samples consisted of a subset of participants from the larger ROS autopsy study. Dementia status was determined at each study visit by trained clinicians using all cognitive and clinical data blinded to prior years and based on NINCDS-ADRDA recommendations. For participants diagnosed with AD, “age at first AD diagnosis”, the best approximation of the age of onset, was defined as the age at the visit where an AD diagnosis was rendered. A final consensus clinical diagnosis was determined at death blinded to all neuropathologic data.

Autopsies were performed based on standard methods reported previously^69^. Postmortem brains were examined by an expert neuropathologist or trained technician to assess AD pathology. CERAD and Braak criteria were used to assess the severity of AD pathology, as described previously^70^.

ROS autopsy participants were classified into three groups based on the below criteria:

1) AD participants (*n* = 31) had a final consensus clinical diagnosis of AD and an NIA-Reagan score of intermediate or high likelihood of AD. NIA-Reagan criteria are based on both neuritic plaques (CERAD score) and neurofibrillary tangles (Braak score)^71^.

2) CN participants (*n* = 22) had a clinical diagnosis of no cognitive impairment (NCI) and an NIA-Reagan score of low likelihood or no AD) and therefore free of significant AD-specific pathology at death and cognitive impairment during life.

3) ASY participants (*n* = 18) had a final consensus clinical diagnosis of NCI and an NIA-Regan score of intermediate or high likelihood of AD. Table 1 describes the demographic characteristics of the ROS sample.

All ROS participants provided written informed consent and the study was approved by an Institutional Review Board of Rush University Medical Center. Participants signed an Anatomical Gift Act for organ donation and a repository consent to allow their data and biospecimens to be shared.

### Participants (AD and CN): Gene Expression Omnibus (GEO)

We accessed gene expression data on AD and CN samples from publicly available microarray datasets (Gene Expression Omnibus (GEO): GSE48350 and GSE5281). Both datasets included gene expression data acquired on the Affymetrix U133 Plus2 array platform from 23 brain regions. We analyzed the data from the entorhinal cortex (ERC; AD: *n* = 25 and CN: *n* = 52), hippocampus (AD: *n* = 29 and CN: *n* = 56), and visual cortex (used as a control region) (AD: *n* = 18 and CN: *n* = 12).

GEO data were used for analyses of regional differential brain gene expression and for genome-scale metabolic network modeling described below in the “Statistical analyses” section. For both analyses, we reported pooled results combining both GEO datasets.

### Participants (PD and CN): Gene Expression Omnibus (GEO)

We accessed gene expression data on Parkinson’s disease (PD) and CN samples from publicly available microarray datasets (GEO: GSE20292 and GSE20114). Both datasets included gene expression data acquired on the Affymetrix (U133A and U133 Plus2 arrays) platform. We analyzed data from the substantia nigra, the brain region primarily impacted by PD-specific neuropathology^72^ (PD: *n* = 21 and CN: *n* = 26).

Similar to analyses in AD vs CN, we used GEO data for regional brain gene expression and for genome-scale metabolic network modeling and reported pooled results combining both GEO datasets.

### Brain tissue processing

For brain tissue samples in both BLSA and ROS, we performed targeted metabolomics on two a priori specified regions: the inferior temporal gyrus (ITG) and the middle frontal gyrus (MFG). These two regions were selected as regions vulnerable to β-amyloid and tau deposition, respectively^73,74^. Sample extraction and storage have been described previously^75^.

Brain tissue samples (up to 80 mg) were homogenized with 85/15 ethanol phosphate buffer 1:3 (mg tissue/µL solvent w/v) using a Precellys (4 °C, nitrogen-cooled, with 1.4-mm ceramic beads in 0.5-mL precellys vials, program: 5800 rpm, three cycles each 30 s, 30 s pause) device and centrifuged (10.000 rcf, 2 min, 4 °C). In total, 20 µL sample homogenate supernatant was placed on the 96-well plate Biocrates kit filter plate with prior placed oxysterol-specific stable isotope-labeled internal standards (10 µL, in MeOH + 0.01% butylated hydroxytoluene (BHT), concentration range 0.5–100 µM), dried under nitrogen for 5 min. In all, 14 d6 or d7 deuterium-labeled internal standards appropriate to each of the 14 analytes were used. Free oxysterols were extracted from the sample homogenate supernatant (dried for 30 min under nitrogen) with 100 µL methanol +0.01% BHT by filter plate shaking (20 min at 600 rpm) and centrifugation (2 min at 500 rcf, 4 °C) into the capture plate. 30 µL Milli-Q water was added to each sample extract and carefully shaken for 5 min at 500 rpm.

### Targeted metabolomics

Ultrahigh-Performance Liquid Chromatography-tandem mass spectrometry (UHPLC–MS/MS) with multiple reaction monitoring (MRM)-based detection in positive mode using a SCIEX API 5500 QTRAP^®^ (AB SCIEX, Darmstadt, Germany) instrument with electrospray ionization (ESI) were used for free oxysterol assays. The 96-well plate allows the analysis of up to 75 samples, 1 blank sample, 3 zero samples (internal standards plus extraction solvent), calibrators 1–7, and three quality control levels (QC, low, medium, high in replicates) of human plasma-based materials. Quantitation was performed with deuterium-labeled internal standards for each analyte (mix produced based on Avanti Polar Lipids standards) and 7-point external calibration. The individual calibrators for each analyte are designed in relation to their physiological ranges. Supplementary Table 6 includes quantitation ranges for each metabolite for calibrators 1–7. The assay has been validated according to European Medicines Agency (EMA) guidelines^76^. Analytical intra- and interday/batch precision expressed by the coefficients of variation (CV) using this methodology were <15% (intra-/interday/batch) for all analytes. Batch effects were controlled and adjusted for using MetIDQ software-implemented normalization procedure.

We measured metabolite concentrations across three categories related to cholesterol homeostasis^21^. All metabolites met the inclusion criteria described below (“Statistical analysis”).

1. De novo cholesterol biosynthesis: 24,25-dihydrolanosterol, 7-dehydrocholesterol, desmosterol, lanosterol, and free cholesterol.
2. Cholesterol catabolism (enzymatic): 27-hydroxycholesterol, 4β-hydroxycholesterol, 24S-hydroxycholesterol, and 7α-hydroxycholesterol*.
3. Cholesterol catabolism (non-enzymatic): 5α,6α-epoxycholesterol, 5α,6β-dihydroxycholestanol, 5β,6β-epoxycholesterol, 7-ketocholesterol, and 7β-hydroxycholesterol.

*7α-hydroxycholesterol can be generated both enzymatically and nonenzymatically^77^.

### Cognitive status

In BLSA, evaluation of cognitive status including dementia diagnosis has been described in detail previously^64^.

In ROS, evaluation of cognitive status including dementia diagnosis has been described in detail previously^66,78,79^.

### Regional brain gene expression

We first identified an a priori list of genes known to encode enzymes across three categories related to cholesterol homeostasis:

1. De novo cholesterol biosynthesis
2. Cholesterol catabolism (enzymatic): representing oxysterol biosynthesis from the enzymatic conversion of cholesterol
3. Cholesterol esterification

We examined differential gene expression of these genes in AD vs CN samples in three brain regions. We chose the hippocampus and ERC as the accumulation of pathology in these regions is thought to trigger the onset of AD symptoms^80–82^. We chose the visual cortex as a control region. We tested for differential gene expression in an a priori list of 31 genes known to encode enzymes regulating cholesterol biosynthesis, catabolism (enzymatic), and esterification reactions. Expression levels of these genes were also used in genome-scale metabolic network modeling using Integrative Metabolic Analysis Tool (iMAT)^83^ (described in the “Statistical analysis” section below).

We then examined differential gene expression in the substantia nigra of PD compared CN samples. This analysis was restricted to genes that were significantly differentially expressed in the AD compared CN samples with the goal of testing whether gene expression differences in AD were disease-specific or related to non-specific changes associated with neurodegeneration. Expression levels of these genes in the substantia nigra in PD compared to CN samples were also used in genome-scale metabolic network modeling using iMAT. These analyses were also restricted to reactions that were significantly less active or more active in AD compared CN in the ERC, hippocampus, or visual cortex and tested whether metabolic reactions predicted to be altered in AD were also altered in PD compared to CN samples in the substantia nigra.

### Statistical analyses

Metabolites with >30% of values missing were excluded from all analyses. This threshold is consistent with our prior studies^84,85^. Values that were indicated as less than the limit of detection (LOD) were imputed as the LOD threshold value divided by 2. Because missing values indicated as less than LOD ( < LOD) are not missing at random (NMAR), we used metabolite-specific LOD threshold data to impute a value for metabolites that had < =30% of values missing. We have included the percentage of missing values by brain region across metabolites as well as metabolites that were excluded from analyses based on the 30% threshold in Supplementary Table 7.

For statistical tests, we used an alpha-level of 0.05 as the threshold for statistical significance. Each metabolite tested in this study represented a hypothesis developed a priori based on its established role in specific biochemical pathways as well as a priori-defined brain regions vulnerable to AD pathology.

We first tested for differences in age at death, sex, race, *APOE* genotype, statin use, CERAD scores, Braak scores, and postmortem interval (PMI) across AD, CN, and ASY groups within studies as well as across studies (i.e., BLSA compared to ROS).

Second, in primary analyses, we tested whether brain tissue concentrations of metabolites differed across AD, CN, and ASY groups (i.e., disease status) and were associated with severity of AD pathology (i.e., CERAD and Braak scores) in the ITG and MFG. For models with AD pathology, we examined the association between metabolite levels and CERAD and Braak scores independent of disease status (i.e., disease status was not considered in models). We first visualized linear associations between metabolite concentrations and our predictors of interest: disease status (AD, CN, ASY) (Supplementary Fig. 1) and pathology (CERAD and Braak scores) (Supplementary Figs. 2 and 3) in BLSA and ROS separately. Convergent associations—i.e., where linear associations between metabolite concentration and disease status/ pathology in ROS and BLSA were in a similar direction—were pooled and are presented as primary results (indicated with a “*” in Supplementary Figs. 1–3). As these results represent convergent associations in two independent cohorts, we report significant associations where *P* < 0.05. Divergent associations—i.e., where linear associations between metabolite concentration and disease status/ pathology in ROS and BLSA were in a different direction—were not pooled and are included as cohort-specific secondary analyses in Supplementary Tables. As these secondary results represent divergent associations in cohort-specific models, we report significant associations using the Benjamini–Hochberg false discovery rate (FDR) < 0.05^86^ to correct for the total number of metabolite comparisons within each a priori specified biochemical pathway/cluster.

Similar to our previous metabolomics analyses^84^, in order to test for differences in metabolite concentrations by disease status in the ITG and the MFG, we used linear mixed-effects models in each of the three a priori-defined biochemical pathways (i.e., clusters): de novo cholesterol biosynthesis, cholesterol catabolism (enzymatic), and cholesterol catabolism (non-enzymatic). Log2-transformed metabolite concentration was used as the dependent variable, disease status (i.e., AD, CN, ASY) as the main fixed effect, sex, and age at death as covariates, within-subject covariance structure was modeled as unstructured, and variance was estimated using Huber-White robust variance estimates. We used the same approach to model CERAD and Braak pathology scores substituting pathology for disease status in the model. Significant associations are indicated in Table 2. In Fig. 2, we also visualize significant associations: metabolites highlighted in green indicate that lower metabolite concentration is significantly associated with AD, higher neuritic plaque burden (CERAD score), or higher neurofibrillary tangle pathology (Braak score). Metabolites highlighted in red indicate that higher metabolite concentration is significantly associated with AD, higher neuritic plaque burden (CERAD score), or higher neurofibrillary tangle pathology (Braak score).

For brain gene expression data, we pooled both AD vs CN GEO datasets (GSE48350 and GSE5281) and first normalized the samples using Robust Multi-array Average (RMA)^87^ with the Brainarray ENTREZG (version 22) custom CDF^88^. In order to test for differences between AD and CN in the pooled GEO datasets, we used the R package *limma*^89^ to test each gene univariately, controlling for sex, age, and batch. We used FDR^86^ (*P* < 0.05) to adjust for multiple comparisons accounting for all 20,414 genes on the Affymetrix U133 Plus2.0 array used in both GEO datasets. We highlighted significant (FDR-corrected) genes that were differentially expressed in AD vs CN samples across all three brain regions: hippocampus, ERC, and visual cortex (control region). In a heatmap (Fig. 1), we visualized significant results: red represents increased expression and green represents reduced expression in AD vs CN.

We performed similar analyses for brain gene expression data from the substantia nigra comparing PD vs CN using GEO datasets GSE20292 and GSE20141; Brainarray ENTREZG (version 24) was used to normalize samples. The goal of this analysis was to test whether differential gene expression observed in AD was similar in a non-AD neurodegenerative disease. We, therefore, restricted these analyses to significant genes that were differentially expressed in AD vs CN analyses. We used identical analyses (e.g., R package *limma*^89^ and FDR correction) to test for differences between PD and CN samples, controlling for batch. As one of the PD datasets analyzed (GSE20141) did not include sex or age information, these covariates were not included in this analysis.

Using regional brain gene expression data, we additionally performed genome-scale metabolic network modeling, a computational framework to predict fluxes through multiple metabolic reactions^90,91^. We used the most recent version of the human genome-scale metabolic model (GEM) network, Human-GEM 1.3.2^92^ to create personalized metabolic networks for all (1) AD vs CN samples from the hippocampus, ERC, and primary visual cortex regions across the GSE5281 and GSE48350 datasets; and (2) PD vs CN samples from the substantia nigra across the GSE20292 and GSE20141datasets.

**Fig. 3:**
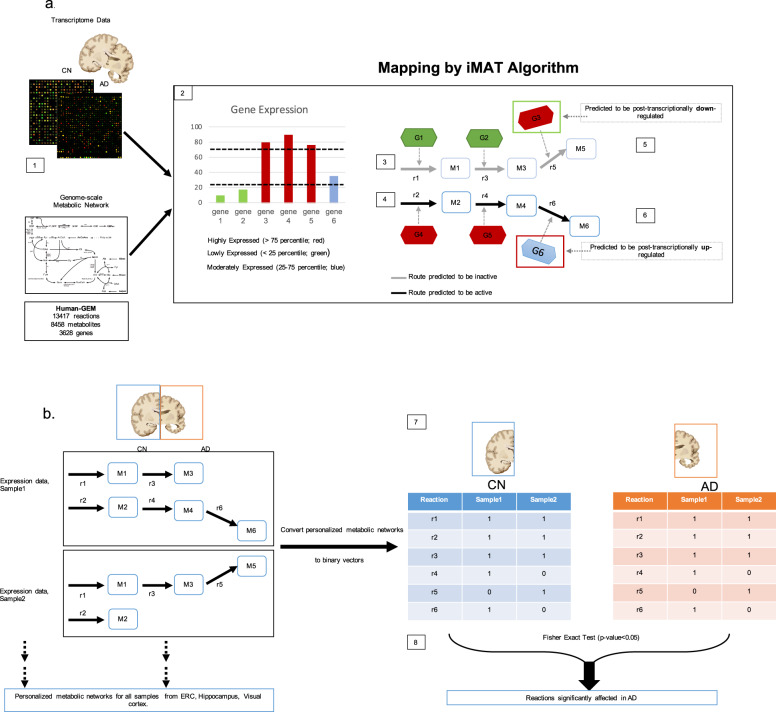
Workflow of iMAT-based metabolic network modeling. AD Alzheimer’s disease, CN control, ERC entorhinal cortex. Description of workflow of iMAT-based metabolic network modeling to predict significantly altered enzymatic reactions relevant to de novo cholesterol biosynthesis, catabolism, and esterification in the AD brain. **a** Our human GEM network included 13417 reactions associated with 3628 genes ([1]). Genes in each sample are divided into three categories based on their expression: highly expressed (>75th percentile of expression), lowly expressed (<25th percentile of expression), or moderately expressed (between 25th and 75th percentile of expression) ([2]). Only highly- and lowly expressed genes are used by iMAT algorithm to categorize the reactions of the Genome-Scale Metabolic Network (GEM) as active or inactive using an optimization algorithm. Since iMAT is based on the prediction of mass-balanced based metabolite routes, the reactions indicated in gray are predicted to be inactive ([3]) by iMAT to ensure maximum consistency with the gene expression data; two genes (G1 and G2) are lowly expressed, and one gene (G3) is highly expressed and therefore considered to be post-transcriptionally downregulated to ensure an inactive reaction flux ([5]). The reactions indicated in black are predicted to be active ([4]) by iMAT to ensure maximum consistency with the gene expression data; 2 genes. (G4 and G5) are highly expressed and one gene (G6) is moderately expressed and therefore considered to be *post-transcriptionally upregulated* to ensure an active reaction flux ([6]). **b** Reaction activity (either active (1) or inactive (0) is predicted for each sample in the dataset ([7]). This is represented as a binary vector that is brain region and disease-condition specific; each reaction is then statistically compared using a Fisher Exact Test to determine whether the activity of reactions is significantly altered between AD and CN samples ([8]).

The human GEM is a stoichiometry-based and mass-balanced computational reconstruction of all known biochemical reactions within human cells. This system-level reconstruction of metabolic processes provides a mechanistic link between genotype and phenotype^93^. This is achieved by writing mass balances around each intracellular metabolite considering the rates (fluxes) of reactions that consume and produce that metabolite^94^. The mass balances are solved as a set of linear equations through optimization to predict fluxes through model reactions in a given condition. Subcellular localization (cytoplasm, mitochondria, endoplasmic reticulum, etc.) is also taken into account in this approach for enzymes located in multiple cellular compartments by defining separate reactions for each compartment. Our human GEM network included 13417 reactions associated with 3628 genes (Fig. 3a[1]). We focused specifically on reactions associated with the a priori list of 31 genes known to encode enzymes regulating de novo cholesterol biosynthesis, catabolism (enzymatic), and esterification reactions (described above in the “Regional brain gene expression” section). We identified a total of 177 reactions controlled by these genes within de novo cholesterol biosynthesis (93 reactions), cholesterol catabolism (enzymatic) (18 reactions), and cholesterol esterification (66 reactions) pathways.

The RMA-normalized and sex- and age-corrected transcriptomic data for AD vs CN data were mapped onto the Human-GEM network separately for each sample in the two GEO datasets using Integrative Metabolic Analysis Tool (iMAT)^83^ (Fig. 3). For PD vs CN, sex and age data were not available in one of the PD datasets (GSE20141) and therefore transcriptomic data was only RMA-normalized.

iMAT is one of the commonly preferred algorithms for metabolic network modeling because it does not require any additional information such as the measured rates of some reactions. iMAT uses an optimization algorithm to maximize the consistency between flux activity predictions from the metabolic network and the gene expression data such that reactions associated with highly expressed genes are pushed to carry a non-zero flux (i.e., active) in the metabolic network while reactions associated with lowly expressed genes are pushed to be inactive. Separately for each of the two GEO datasets, gene expression data across all samples were combined to identify the 25th and 75th percentiles of expression. These were used to determine whether the expression of a specific gene within any given sample was either lowly expressed (i.e., below the 25th percentile), highly expressed (i.e., above the 75th percentile) or moderately expressed (i.e., above the 25th percentile and below the 75th percentile). Only genes that were lowly or highly expressed contributed to iMAT predictions of whether a reaction was transcriptionally downregulated (i.e., inactive) or upregulated (i.e., active) (Fig. 3a[2]).

In mapping the expression data on reactions associated with multiple genes, the maximum gene expression value among the multiple genes was assigned to that reaction for isoenzymes (where multiple genes can independently code for enzymes that catalyze a reaction), and the minimum gene expression value among the multiple genes was assigned to that reaction for enzyme complexes (where multiple genes are required to code for an enzyme)^90^. Three rate constraints were introduced in the iMAT simulations of both AD and CN samples (or PD and CN samples) to ensure their activity: glucose and oxygen uptake rates and active macromolecule synthesis rate had their lower bounds set to 0.01, 0.01, and 0.0001, respectively, leaving all the other reaction rates unconstrained in the simulations. Therefore, these three reactions were always active in all personalized models generated and differences in the predicted reaction activities between AD and CN (or PD and CN) were only due to differences in the set of highly and lowly expressed reactions for each sample as well as the consequent change in the mass-balance-based metabolite routes in the network.

After reactions were predicted by iMAT as either inactive or active, within each disease group (AD and CN) Fig. 3b[6]), each sample was represented as a binary vector (active = 1; inactive = 0) for each reaction (Fig. 3b[7]). The group and region-specific binary vectors were then compared using the Fisher Exact Test to determine whether the activity of reactions (active or inactive) were significantly (*P* < 0.05) altered between AD and CN (Fig. 3b[8])^57^. We indicated significant results in the hippocampus and ERC as well as the visual cortex (control region). We performed similar analyses in PD compared to CN samples in the substantia nigra. The goal of this analysis was to test whether reactions that were significantly altered in AD were similarly altered in a non-AD neurodegenerative disease. We, therefore, restricted these analyses to reactions that were significantly less active or more active in AD compared to CN in the ERC, hippocampus, or visual cortex.

Simulations were performed in MATLAB R2018a using Gurobi optimizer and the iMAT implementation available under COBRA Toolbox^95^.

In order to enhance the interpretability of our metabolite, gene expression, and metabolic network modeling results, we visualize results in pathway figures (Fig. 2) including the following categories: de novo cholesterol biosynthesis; cholesterol catabolism (enzymatic); and cholesterol esterification.

### Reporting summary

Further information on research design is available in the Nature Research Reporting Summary linked to this article.

## Supplementary information

Reporting Summary

Supplementary Information

Supplementary Data 1

## Data Availability

Data from the Baltimore Longitudinal Study of Aging (BLSA) are available to researchers and can be requested at https://www.blsa.nih.gov/researchers. Data from the Religious Orders Study (ROS) can be requested by researchers at www.radc.rush.edu. Gene Expression Omnibus (GEO) data is publicly available at https://www.ncbi.nlm.nih.gov/geo/ and includes GEO ascension numbers GSE48350, GSE5281, GSE20292, and GSE20141.

## Code availability

STATA 15.1, R 3.5.1, and MATLAB R2018a were used for all analyses. Code used to analyze results can be requested from the corresponding author. All codes used for analyses in this study are available to researchers and can be requested by contacting the corresponding author.
