# Supplementary material for: Abnormal brain cholesterol homeostasis in Alzheimer’s disease—a targeted metabolomic and transcriptomic study
Source: NPJ Aging Mech Dis. 2021 Jun 1;7:11. doi: 10.1038/s41514-021-00064-9 (PMC8169871; doi:10.1038/s41514-021-00064-9)
Supplement: Supplementary file 1 — Reporting Summary [file 41514_2021_64_MOESM1_ESM.pdf]

Corresponding author(s): Madhav Thambisetty

Last updated by author(s): Mar 4, 2021

Reporting Summary

Nature Research wishes to improve the reproducibility of the work that we publish. This form provides structure for consistency and transparency in reporting. For further information on Nature Research policies, see our Editorial Policies and the Editorial Policy Checklist.

Statistics

For all statistical analyses, confirm that the following items are present in the figure legend, table legend, main text, or Methods section.

n/a	Confirmed	
☐	☒	The exact sample size (*n*) for each experimental group/condition, given as a discrete number and unit of measurement
☐	☒	A statement on whether measurements were taken from distinct samples or whether the same sample was measured repeatedly
☐	☒	The statistical test(s) used AND whether they are one- or two-sided<br>*Only common tests should be described solely by name; describe more complex techniques in the Methods section.*
☐	☒	A description of all covariates tested
☐	☒	A description of any assumptions or corrections, such as tests of normality and adjustment for multiple comparisons
☐	☒	A full description of the statistical parameters including central tendency (e.g. means) or other basic estimates (e.g. regression coefficient) AND variation (e.g. standard deviation) or associated estimates of uncertainty (e.g. confidence intervals)
☐	☒	For null hypothesis testing, the test statistic (e.g. *F*, *t*, *r*) with confidence intervals, effect sizes, degrees of freedom and *P* value noted<br>*Give P values as exact values whenever suitable.*
☒	☐	For Bayesian analysis, information on the choice of priors and Markov chain Monte Carlo settings
☒	☐	For hierarchical and complex designs, identification of the appropriate level for tests and full reporting of outcomes
☐	☒	Estimates of effect sizes (e.g. Cohen's *d*, Pearson's *r*), indicating how they were calculated

Our web collection on statistics for biologists contains articles on many of the points above.

Software and code

Policy information about availability of computer code

Data collection	Metabolomics software used by Biocrates: Batch effects were controlled and adjusted for using MetIDQ software-implemented normalization procedure.
Data analysis	STATA 15.1, R 3.5.1, and MATLAB R2018a were used for all analyses. Code used to analyze results can be requested from the corresponding author.

For manuscripts utilizing custom algorithms or software that are central to the research but not yet described in published literature, software must be made available to editors and reviewers. We strongly encourage code deposition in a community repository (e.g. GitHub). See the Nature Research guidelines for submitting code & software for further information.

Data

Policy information about availability of data

All manuscripts must include a data availability statement. This statement should provide the following information, where applicable:

- Accession codes, unique identifiers, or web links for publicly available datasets
- A list of figures that have associated raw data
- A description of any restrictions on data availability

Data from the Baltimore Longitudinal Study of Aging (BLSA) are available to researchers and can be requested at https://www.blsa.nih.gov/researchers. Data from the Religious Orders Study (ROS) can be requested by researchers at www.radc.rush.edu. Gene Expression Omnibus (GEO) data is publicly available at https://www.ncbi.nlm.nih.gov/geo/.

Field-specific reporting

Please select the one below that is the best fit for your research. If you are not sure, read the appropriate sections before making your selection.

☒ Life sciences ☐ Behavioural & social sciences ☐ Ecological, evolutionary & environmental sciences

For a reference copy of the document with all sections, see nature.com/documents/nr-reporting-summary-flat.pdf

Life sciences study design

All studies must disclose on these points even when the disclosure is negative.

Sample size	Sample size was chosen based on bio-repository or public data repository availability based on specified inclusion/ exclusion criteria
Data exclusions	BLSA and ROS participants from the autopsy program that could not be classified as having a clinical diagnosis of AD/ MCI during life and AD path at autopsy were excluded from the AD group. Participants that could not be classified as cognitively normal during life and free of AD pathology at death were excluded from the AD group. Participants that could not be classified as cognitively normal during life and having AD pathology at death were excluded from the ASY group.<br><br>GEO data for AD and CN samples that did not include AD cases and at least one of the specific brain regions included in the study (ERC, hippocampus, visual cortex) were excluded. GEO data for PD and CN samples that did not include PD cases and at least one of the specific brain regions included in the study (substantia nigra) were excluded.
Replication	We explored associations between brain tissue metabolites and disease status and AD pathology in two distinct study populations that differ across demographic and biologic characteristics. We pooled data across both cohorts and only reported converging results which suggest that findings replicate across cohorts and likely reflect fundamental features of AD pathogenesis.
Randomization	Participants were not randomized to disease groups. In BLSA and ROS, disease groups were determined based on cognitive and pathology criteria. Disease group for participants included in GEO data were determined based on ADRC/ADC/ Brain Bank criteria.
Blinding	Investigators were not blinded to disease status however, disease status was not determined solely by investigators and was determined by a consensus diagnosis conference.

Reporting for specific materials, systems and methods

We require information from authors about some types of materials, experimental systems and methods used in many studies. Here, indicate whether each material, system or method listed is relevant to your study. If you are not sure if a list item applies to your research, read the appropriate section before selecting a response.

Materials & experimental systems

n/a	Involved in the study	
☒	☐	Antibodies
☒	☐	Eukaryotic cell lines
☒	☐	Palaeontology and archaeology
☒	☐	Animals and other organisms
☐	☒	Human research participants
☒	☐	Clinical data
☒	☐	Dual use research of concern

Methods

n/a	Involved in the study	
☒	☐	ChIP-seq
☒	☐	Flow cytometry
☒	☐	MRI-based neuroimaging

Human research participants

Policy information about studies involving human research participants

Population characteristics	For BLSA, participants had an average age at death of 87 years, were 48% female, 93% white, and 31% APOE4 carrier. For ROS, participants had an average age at death of 90 years, were 77% female, 100% white, and 28% APOE4 carrier. GEO participant characteristics have been described in detail in the index studies (see GEO ascension numbers).
Recruitment	BLSA participants are from the autopsy sample of the BLSA, a prospective cohort study administered by the National Institute on Aging. Autopsy samples were similar demographically to the larger study; recruitment has been described previously. ROS participants are from the ROS study, a longitudinal, clinical and pathologic cohort study of individuals within religious communities across the U.S. Both BLSA and ROS participants are not representative of the general population. Recruitment for GEO participants have been described in detail in the index studies (see GEO ascension numbers).
Ethics oversight	The BLSA study protocol has ongoing approval from the Institutional Review Board of the National Institute of Environmental Health Science, National Institutes of Health. Written informed consent was obtained at each visit from all participants. All ROS participants provided written informed consent and the study was approved by an Institutional Review Board of Rush University Medical Center. Participants signed an Anatomical Gift Act for organ donation and a repository consent to allow their data and biospecimens to be shared. Ethics oversight for GEO participants have been described in detail in teh index studies (see GEO ascension numbers).

Note that full information on the approval of the study protocol must also be provided in the manuscript.
