# Supplementary material for: Abnormal brain cholesterol homeostasis in Alzheimer’s disease—a targeted metabolomic and transcriptomic study
Source: NPJ Aging Mech Dis. 2021 Jun 1;7:11. doi: 10.1038/s41514-021-00064-9 (PMC8169871; doi:10.1038/s41514-021-00064-9)
Supplement: Supplementary file 2 — Supplementary Information [file 41514_2021_64_MOESM2_ESM.pdf]

Supplementary Table 1. Cohort-specific associations between metabolite levels and disease status/ severity of AD pathology

	BLSA						ROS					
	ITG			MFG			ITG			MFG		
Metabolite Name	β	p-value	p-value (FDR)	β	p-value	p-value (FDR)	β	p-value	p-value (FDR)	β	p-value	p-value (FDR)
**Disease Status**												
**De novo cholesterol biosynthesis**												
cholesterol	-0.063	0.44	0.915	-0.019	0.836	0.836	0.076	0.055	0.25	0.024	0.611	0.719
lanosterol	0.016	0.915	0.915	-0.204	0.133	0.332	-0.127	0.183	0.25	**-0.217**	**0.019**	**0.093**
24,25-dihydrolanosterol	-0.045	0.699	0.915	0.126	0.298	0.497	0.067	0.254	0.254	-0.022	0.719	0.719
7-dehydrocholesterol	-0.387	0.172	0.86	**-0.499**	**0.034**	**0.169**	0.391	0.14	0.25	0.512	0.101	0.189
desmosterol	-0.075	0.823	0.915	0.058	0.752	0.836	-0.335	0.2	0.25	-0.336	0.114	0.189
**Cholesterol catabolism (enzymatic)**												
27-hydroxycholesterol	0.406	0.143	0.191	0.185	0.539	0.539	0.186	0.22	0.321	**-0.345**	**0.049**	**0.065**
4β-hydroxycholesterol	0.1	0.61	0.61	0.242	0.113	0.452	0.107	0.321	0.321	-0.111	0.285	0.285
24S-hydroxycholesterol	**-0.236**	**0.022**	**0.089**	0.101	0.376	0.502	-0.054	0.214	0.321	**-0.15**	**0.009**	**0.036**
7α-hydroxycholesterol	0.23	0.124	0.191	0.145	0.371	0.502	0.097	0.308	0.321	**-0.202**	**0.024**	**0.048**
**Cholesterol catabolism (non-enzymatic)**												
5α,6α-epoxycholesterol	0.096	0.561	0.561	0.134	0.383	0.66	0.157	0.14	0.159	0.067	0.421	0.526
5α,6β-dihydroxycholestanol	0.128	0.378	0.472	0.029	0.883	0.883	**0.221**	**0.02**	**0.054**	**-0.229**	**0.016**	**0.039**
5β,6β-epoxycholesterol	0.126	0.354	0.472	0.137	0.396	0.66	0.146	0.089	0.149	0.005	0.943	0.943
7-ketocholesterol	**0.282**	**0.019**	**0.096**	0.073	0.563	0.704	**0.22**	**0.022**	**0.054**	-0.123	0.113	0.188
7β-hydroxycholesterol	**0.338**	**0.042**	**0.106**	0.278	0.233	0.66	0.129	0.159	0.159	**-0.211**	**0.014**	**0.039**
**CERAD**												
**De novo cholesterol biosynthesis**												
cholesterol	-0.043	0.357	0.801	-0.024	0.752	0.94	0.032	0.275	0.412	0.009	0.786	0.983
lanosterol	0.004	0.969	0.969	-0.119	0.258	0.634	-0.069	0.261	0.412	-0.097	0.136	0.227
24,25-dihydrolanosterol	-0.042	0.608	0.801	0.088	0.38	0.634	0.019	0.644	0.644	-0.001	0.983	0.983
7-dehydrocholesterol	-0.305	0.136	0.68	-0.307	0.082	0.411	**0.488**	**0.01**	**0.05**	**0.559**	**0.016**	**0.082**
desmosterol	-0.122	0.64	0.801	0.002	0.984	0.984	-0.182	0.329	0.412	-0.303	0.052	0.131
**Cholesterol catabolism (enzymatic)**												

27-hydroxycholesterol	0.157	0.471	0.628	-0.015	0.944	0.944	0.037	0.722	0.931	-0.174	0.119	0.119
4β-hydroxycholesterol	0.065	0.647	0.647	0.164	0.202	0.624	-0.007	0.931	0.931	**-0.186**	**0.007**	**0.029**
24S-hydroxycholesterol	**-0.168**	**0.011**	**0.042**	0.035	0.666	0.888	-0.048	0.083	0.214	-0.075	0.056	0.075
7α-hydroxycholesterol	0.156	0.154	0.308	0.12	0.312	0.624	0.103	0.107	0.214	**-0.128**	**0.019**	**0.037**
**Cholesterol catabolism (non-enzymatic)**												
5α,6α-epoxycholesterol	0.05	0.695	0.699	0.106	0.357	0.649	0.096	0.262	0.262	0.006	0.927	0.927
5α,6β-dihydroxycholestanol	0.042	0.699	0.699	-0.066	0.661	0.67	0.105	0.139	0.262	-0.127	0.088	0.147
5β,6β-epoxycholesterol	0.079	0.462	0.699	0.106	0.39	0.649	0.079	0.254	0.262	-0.03	0.593	0.741
7-ketocholesterol	0.152	0.086	0.216	0.036	0.67	0.67	0.138	0.051	0.253	**-0.111**	**0.038**	**0.094**
7β-hydroxycholesterol	0.222	0.08	0.216	0.236	0.201	0.649	0.091	0.185	0.262	**-0.14**	**0.019**	**0.094**
**Braak**												
**De novo cholesterol biosynthesis**												
cholesterol	0.001	0.973	0.973	0.03	0.631	0.706	0.044	0.118	0.227	0.051	0.071	0.118
lanosterol	-0.014	0.867	0.973	-0.128	0.121	0.303	**-0.169**	**0.003**	**0.013**	**-0.161**	**0.014**	**0.069**
24,25-dihydrolanosterol	-0.029	0.569	0.973	0.028	0.706	0.706	-0.05	0.2	0.227	-0.019	0.653	0.653
7-dehydrocholesterol	-0.011	0.913	0.973	-0.126	0.186	0.31	0.249	0.157	0.227	0.143	0.532	0.653
desmosterol	0.33	0.192	0.96	0.2	0.025	0.126	-0.161	0.227	0.227	-0.204	0.069	0.118
**Cholesterol catabolism (enzymatic)**												
27-hydroxycholesterol	0.015	0.937	0.937	-0.139	0.493	0.564	0.131	0.216	0.432	-0.057	0.595	0.793
4β-hydroxycholesterol	0.029	0.757	0.937	0.072	0.436	0.564	-0.013	0.819	0.819	-0.014	0.857	0.857
24S-hydroxycholesterol	**-0.112**	**0.033**	**0.13**	-0.065	0.318	0.564	**-0.064**	**0.038**	**0.152**	-0.079	0.1	0.2
7α-hydroxycholesterol	0.01	0.92	0.937	-0.057	0.564	0.564	-0.02	0.806	0.819	**-0.132**	**0.032**	**0.13**
**Cholesterol catabolism (non-enzymatic)**												
5α,6α-epoxycholesterol	-0.009	0.925	0.989	0.004	0.96	0.96	0.092	0.27	0.45	0.024	0.732	0.915
5α,6β-dihydroxycholestanol	-0.001	0.989	0.989	-0.125	0.3	0.928	**0.094**	**0.048**	**0.242**	-0.087	0.094	0.245
5β,6β-epoxycholesterol	0.014	0.869	0.989	-0.037	0.712	0.928	0.076	0.263	0.45	-0.001	0.989	0.989
7-ketocholesterol	0.044	0.585	0.989	-0.029	0.67	0.928	0.072	0.398	0.498	-0.057	0.392	0.653
7β-hydroxycholesterol	0.035	0.76	0.989	-0.05	0.742	0.928	0.01	0.896	0.896	-0.13	0.098	0.245

Inferior Temporal Gyrus (ITG); Middle Frontal Gyrus (MFG); False Discovery Rate (FDR); Baltimore Longitudinal Study on Aging (BLSA); Religious Orders Study (ROS); $p < 0.05$ in bold

Negative coefficients indicate that lower metabolite concentration is significantly associated with AD, higher neuritic plaque burden (CERAD score), or higher neurofibrillary tangle pathology (Braak score). Positive coefficients indicate that higher metabolite concentration is significantly associated with AD, higher neuritic plaque burden (CERAD score), or higher neurofibrillary tangle pathology (Braak score). Metabolite categories are indicated in bold. BLSA sample size: AD (n=15), CN (n=8); ROS sample size: AD (n=31), CN (n=22).

Supplementary Table 2. Differential expression (AD versus CN) of genes regulating *de novo* cholesterol biosynthesis, enzymatic catabolism and esterification within the entorhinal cortex, hippocampus and visual cortex

	entorhinal cortex		hippocampus		visual cortex	
Gene	log fold-change	P-value (FDR)	log fold-change	P-value (FDR)	log fold-change	P-value (FDR)
**De novo Cholesterol biosynthesis**						
ACAT1	0.072	0.440	-0.104	0.229	-0.165	0.280
ACAT2	-0.349	**0.008**	-0.665	**0.000**	-0.457	0.183
CYP51A1	-0.216	0.069	0.064	0.606	-0.562	0.054
DHCR24	-0.599	**0.003**	-0.878	**0.000**	-0.574	0.117
DHCR7	-0.040	0.649	-0.179	0.054	0.130	0.416
FDFT1	-0.081	0.510	-0.045	0.683	-0.078	0.710
FDPS	-0.301	**0.044**	-0.376	**0.002**	-0.641	0.082
GGPPS1	-0.067	0.435	-0.026	0.717	-0.149	0.436
HMGCR	-0.474	**0.044**	-0.702	**0.000**	-0.846	0.065
HMGCS1	-0.256	0.160	-0.447	**0.000**	-0.202	0.330
IDI1	-0.235	**0.002**	-0.307	**0.000**	-0.271	0.144
IDI2	-0.122	0.110	0.108	0.141	0.288	0.148
LSS	0.171	0.173	0.228	**0.038**	0.250	0.227
MVD	-0.118	0.226	-0.194	**0.003**	0.128	0.644
MVK	0.045	0.538	0.078	0.128	0.192	0.289
PMVK	0.045	0.700	-0.142	0.214	0.025	0.921
SC4MOL	-0.402	0.068	-0.192	0.145	-0.454	0.086
SC5D	-0.217	0.089	-0.156	0.219	-0.618	0.066
SQLE	-0.182	0.082	-0.161	0.117	-0.318	0.096
TM7SF2	-0.510	**0.000**	-0.451	**0.000**	-0.232	0.174
**Cholesterol catabolism (enzymatic)**						
CH25H	0.329	0.061	0.152	0.621	-0.549	0.105
CYP11A1	0.006	0.960	0.253	**0.019**	0.200	0.436
CYP27A1	0.282	0.077	0.209	0.122	0.229	0.305
CYP39A1	0.106	0.174	0.143	0.159	0.389	0.074
CYP3A4	0.226	**0.024**	0.167	**0.027**	0.332	0.179
CYP46A1	-0.640	**0.000**	0.080	0.649	0.270	0.115
CYP7A1	-0.036	0.636	-0.157	**0.038**	0.263	0.134
CYP7B1	0.099	0.396	-0.111	0.363	-0.103	0.409
CYP8B1	0.137	0.125	0.041	0.604	0.273	0.177
HSD3B7	-0.036	0.777	0.217	**0.011**	0.255	0.187
**Cholesterol Esterification**						
SOAT1	0.241	**0.001**	0.121	0.157	0.211	0.129

False Discovery Rate (FDR)

Summary of genes differentially expressed in selected brain regions in AD compared to control (CN) across three pathways (de novo cholesterol biosynthesis, cholesterol catabolism (enzymatic) and cholesterol

esterification). P-values indicated in bold were significant at FDR-corrected p-value < 0.05. Metabolite categories are indicated in bold. Gene Expression Ominbus (GEO) data sample size: ERC: AD (n=25), CN (n=52); hippocampus: AD (n=29), CN (n=56); visual cortex: AD (n=18), CN (n=12).

Supplementary Table 3. Differential expression (PD versus CN) of genes regulating *de novo* cholesterol biosynthesis, enzymatic catabolism and esterification within the substantia nigra

Gene	substantia nigra log fold-change	substantia nigra P-value (FDR)
**De novo Cholesterol biosynthesis**		
DHCR24	0.313	0.609
TM7SF2	-0.034	0.816
HMGCR	-0.481	0.210
ACAT2	-0.483	0.210
FDPS	-0.479	0.457
HMGCS1	-0.510	0.210
IDI1	-0.654	0.210
MVD	0.225	0.416
LSS	0.022	0.816
Cholesterol catabolism (enzymatic)		
CYP7A1	0.104	0.694
CYP46A1	0.125	0.694
HSD3B7	0.365	0.349
CYP11A1	0.158	0.250
CYP3A4	0.249	0.250
Cholesterol esterification		
SOAT1	-0.087	0.609

False Discovery Rate (FDR)

Summary of genes differentially expressed in the substantia nigra in PD compared to control (CN) across three pathways (de novo cholesterol biosynthesis, cholesterol catabolism (enzymatic) and cholesterol esterification). Genes included were those significantly different in AD compared to CN. Note: there were no genes that were significantly different between PD compared to CN at an FDR-corrected p-value < 0.05. Metabolite categories are indicated in bold. Gene Expression Ominbus (GEO) data sample size: substantia nigra: PD (n=21), CN (n=26).

Supplementary Table 4. iMAT-based metabolic network modeling of cholesterol synthesis and catabolism in AD

[see xls doc]

Supplementary Table 5. iMAT-based metabolic network modeling of cholesterol synthesis and catabolism in PD

			Substantia Nigra	
Gene	Human-GEM rxn ID	GEM Reaction	Odds Ratio	P-value
		***De novo* Cholesterol Biosynthesis (pre-squalene mevalonate pathway)**		
ACAT2	HMR_1434	acetoacetyl-CoA[c] + CoA[c] <=> 2 acetyl-CoA[c]	0.921	1.000
HMGCS	HMR_1437	acetoacetyl-CoA[c] + acetyl-CoA[c] + H2O[c] => CoA[c] + H+[c] + HMG-CoA[c]	1.833	0.375
HMGCR	HMR_1440	2 H+[c] + HMG-CoA[c] + 2 NADPH[c] => (R)-mevalonate[c] + CoA[c] + 2 NADP+[c]	0.750	0.770
		***De novo* Cholesterol Biosynthesis (post-squalene mevalonate pathway including the Bloch and Kandutsch-Russell pathways)**		
SC5D	7DHCHSTEROLtr	7-dehydrocholesterol[r] <=> 7-dehydrocholesterol[c]	1.392	0.767
DHCR7	HMR_1565	H+[c] + NADPH[c] + 7-dehydrocholesterol[c] => cholesterol[c] + NADP+[c]	0.909	1.000
DHCR7	DHCR72r	H+[r] + NADPH[r] + 7-dehydrocholesterol[r] => cholesterol[r] + NADP+[r]	1.239	0.773
SC4MOL, SC5D	C14STRr	H+[r] + NADPH[r] + 4,4-dimethyl-5alpha-cholesta-8,14,24-trien-3beta-ol[r] => NADP+[r] + 14-demethyllanosterol[r]	0.285	0.159
SC4MOL	C4STMO2Pr	NADP+[r] + O2[r] + 3-keto-4-methylzymosterol[r] => CO2[r] + H+[r] + NADPH[r] + zymosterol Intermediate 2[r]	0.703	0.746
SC5D	HMR_1516	5alpha-cholesta-7,24-dien-3beta-ol[c] + H+[c] + NADPH[c] + O2[c] => 7-dehydrodesmosterol[c] + 2 H2O[c] + NADP+[c]	1.060	1.000
SC5D	LSTO1r	H+[r] + NADPH[r] + O2[r] + 5alpha-cholesta-7,24-dien-3beta-ol[r] => 2 H2O[r] + NADP+[r] + 7-dehydrodesmosterol[r]	2.850	0.268
DHCR7	HMR_1519	7-dehydrodesmosterol[c] + H+[c] + NADPH[c] => desmosterol[c] + NADP+[c]	1.060	1.000
DHCR7	DHCR71r	H+[r] + NADPH[r] + 7-dehydrodesmosterol[r] => NADP+[r] + desmosterol[r]	2.850	0.268
DHCR24	HMR_1526	desmosterol[c] + H+[c] + NADPH[c] => cholesterol[c] + NADP+[c]	1.275	1.000
DHCR24	DSREDUCr	H+[r] + NADPH[r] + desmosterol[r] => cholesterol[r] + NADP+[r]	1.275	1.000
DHCR24	DSMSTEROLtr	desmosterol[r] => desmosterol[c]	2.600	0.146
		**Cholesterol Catabolism (enzymatic)**		
HSD3B7	HMR_1738	cholest-5-ene-3beta,7alpha,24(S)-triol[c] + NAD+[c] => 4-cholesten-7alpha,24(S)-diol-3-one[c] + H+[c] + NADH[c]	0.000	0.446

GEM: genome-scale metabolic model; Human-GEM rxn ID: Human-GEM reaction ID is searchable in metabolicatlas.org and indicates the specific reaction equation and additional reaction details; PD: Parkinson’s disease; CN: Control; [c]: cytoplasm; [m]: mitochondria; [r]: endoplasmic reticulum;

Significant ($p < 0.05$) odds ratios less than 1.0 indicate that the reaction is less active in PD compared to CN; significant odds ratios greater than 1.0 indicate that the reaction more active in PD compared to CN. Note: this analysis was restricted to the 16 reactions predicated by iMAT to be altered in AD compared to CN samples (see Table 3); none of these reactions was significantly different between PD compared to CN in the substantia nigra. GEM reaction categories are indicated in bold. Gene Expression Ominbus (GEO) data sample size: substantia nigra: PD (n=21), CN (n=26).

Supplementary Table 6. Quantitation ranges (calibrator 1-7) in µM

metabolite	cal 1	cal 2	cal 3	cal 4	cal 5	cal 6	cal 7
cholesterol	50	125	500	1000	2000	5000	10.000
lanosterol	0.040	0.100	0.398	0.746	1.491	3.728	7.455
24,25-dihydrolanosterol	0.030	0.075	0.302	0.754	1.509	3.772	7.545
7-dehydrocholesterol	0.01	0.025	0.1	1	2	5	10
desmosterol	0.1	0.25	1	2	4	10	20
27-hydroxycholesterol	0.01	0.025	0.1	0.2	0.4	1	2
4ß-hydroxycholesterol	0.01	0.025	0.1	0.4	0.8	2	4
24S-hydroxycholesterol	0.01	0.025	0.1	0.2	0.4	1	2
7α-hydroxycholesterol	0.01	0.025	0.1	0.5	1	2.5	5
5α,6α-epoxycholesterol	0.004	0.011	0.044	0.174	0.349	0.872	1.743
5α,6β-dihydroxycholestanol	0.005	0.014	0.055	0.219	0.438	1.094	2.189
5 β 6 β -epoxycholesterol	0.006	0.014	0.056	0.226	0.451	1.128	2.257
7-ketocholesterol	0.005	0.0125	0.05	0.5	1	2.5	5
7β-hydroxycholesterol	0.01	0.025	0.1	0.5	1	2.5	5

cal: calibtrator

Supplementary Table 7. Percentage of missing values (i.e. < LOD) across cholesterol biosynthesis and catabolism metabolites

	Brain region	
	**ITG**	**MFG**
cholesterol	0	0
lanosterol	0	0
24,25-dihydrolanosterol	0	0
7-dehydrocholesterol	9	14.14
desmosterol	4	2.02
27-hydroxycholesterol	0	0
4ß-hydroxycholesterol	1	0
24S-hydroxycholesterol	0	0
7α-hydroxycholesterol	0	0
5α,6α-epoxycholesterol	0	0
5α,6ß-dihydroxycholestanol	3	4.04
5ß,6ß-epoxycholesterol	0	0
7-ketocholesterol	0	0
7ß-hydroxycholesterol	0	0
*22R-hydroxycholesterol	68	71.72
*24,25-epoxycholesterol	51	62.63
*25-hydroxycholesterol	100	100
*7a-hydroxycholestenone	76	78.79

ITG: inferior temporal gyrus; MFG: middle frontal gyrus; LOD: limit of detection
Metabolites that were above the 30% missing threshold were not included in analyses and indicated with an *. Values for metabolites that were < LOD and below the 30% missing threshold (i.e. metabolites that are not indicated with an *) were imputed. Baltimore Longitudinal Study on Aging (BLSA) sample size: AD (n=15), CN (n=8); Religious Orders Study (ROS) sample size: AD (n=31), CN (n=22).

Supplementary Figure 1. Cohort-specific linear associations between metabolite concentration and disease status

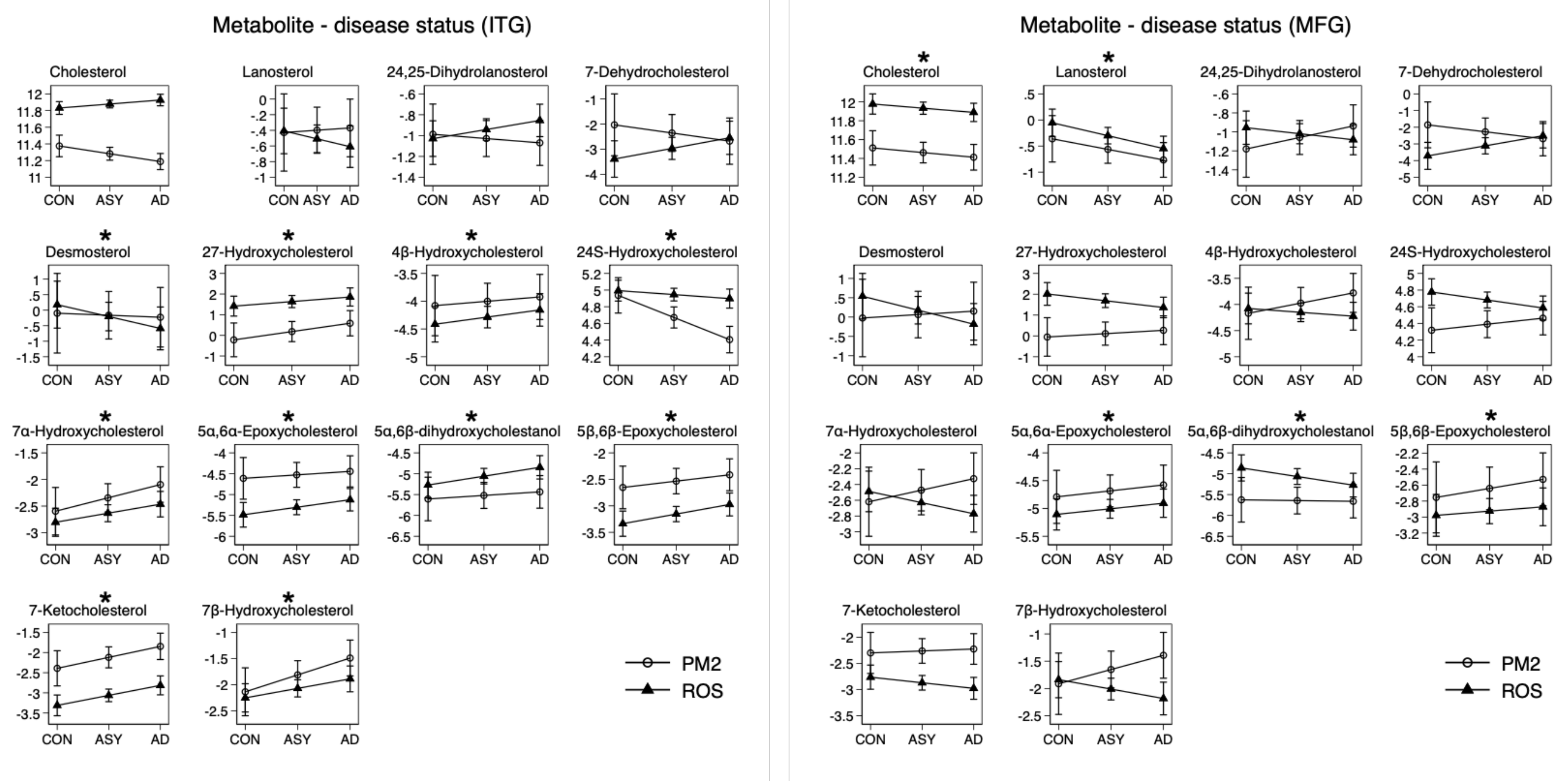

Alzheimer's disease (AD); Cognitively normal (CN); Asymptomatic AD (ASY); Inferior Temporal Gyrus (ITG); Middle Frontal Gyrus (MFG) Convergent associations – where linear associations between metabolite concentration and disease status/ pathology in ROS and BLSA were in a similar direction – were pooled and indicated with a "*". For primary analyses (Table 1), linear mixed effects models were used for each of three a-priori-defined biochemical pathways (i.e. clusters): *de novo* cholesterol biosynthesis, cholesterol catabolism (enzymatic) and cholesterol catabolism (non-enzymatic). At least two metabolites indicated with a * within each cluster were required in order to run models; clusters where only one metabolite could be pooled are presented within cohort (i.e. study) specific secondary analyses (Supplementary Table 1). Error bars indicate 95% confidence intervals. Baltimore Longitudinal Study on Aging (BLSA) sample size: AD (n=15), CN (n=8); Religious Orders Study (ROS) sample size: AD (n=31), CN (n=22).

Supplementary Figure 2. Cohort-specific linear associations between metabolite concentration and neuritic plaques (CERAD score)

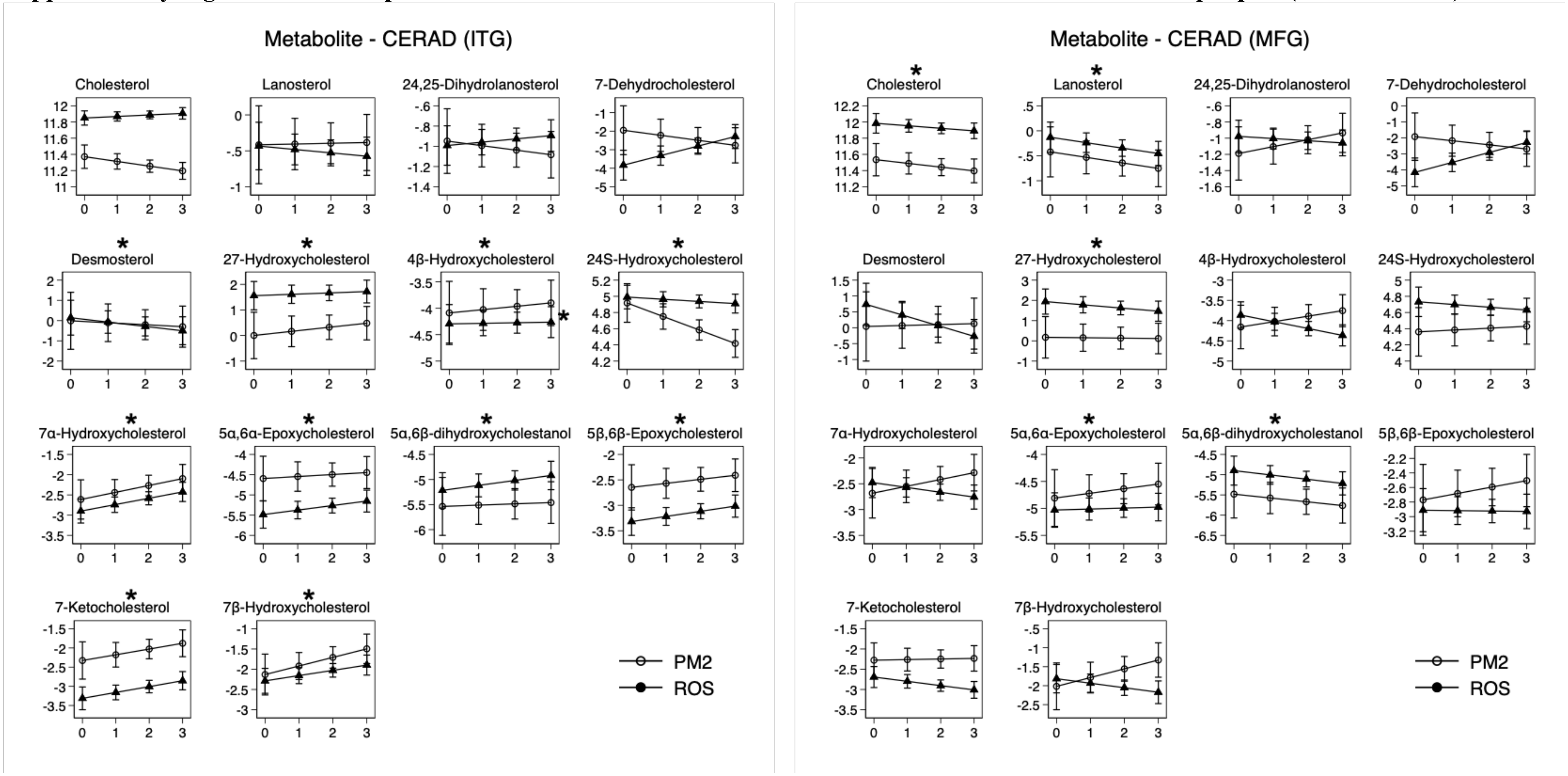

Alzheimer's disease (AD); Cognitively normal (CN); Asymptomatic AD (ASY); Inferior Temporal Gyrus (ITG); Middle Frontal Gyrus (MFG) Convergent associations – where linear associations between metabolite concentration and disease status/ pathology in ROS and BLSA were in a similar direction – were pooled and indicated with a "*". For primary analyses (Table 1), linear mixed effects models were used for each of three a-priori-defined biochemical pathways (i.e. clusters): *de novo* cholesterol biosynthesis, cholesterol catabolism (enzymatic) and cholesterol catabolism (non-enzymatic). At least two metabolites indicated with a * within each cluster were required in order to run models; clusters where only one metabolite could be pooled are presented within cohort (i.e. study) specific secondary analyses (Supplementary Table 1). Error bars indicate 95% confidence intervals. Baltimore Longitudinal Study on Aging (BLSA) sample size: AD (n=15), CN (n=8); Religious Orders Study (ROS) sample size: AD (n=31), CN (n=22).

Supplementary Figure 3. Cohort-specific linear associations between metabolite concentration and neurofibrillary tangle pathology (Braak score)

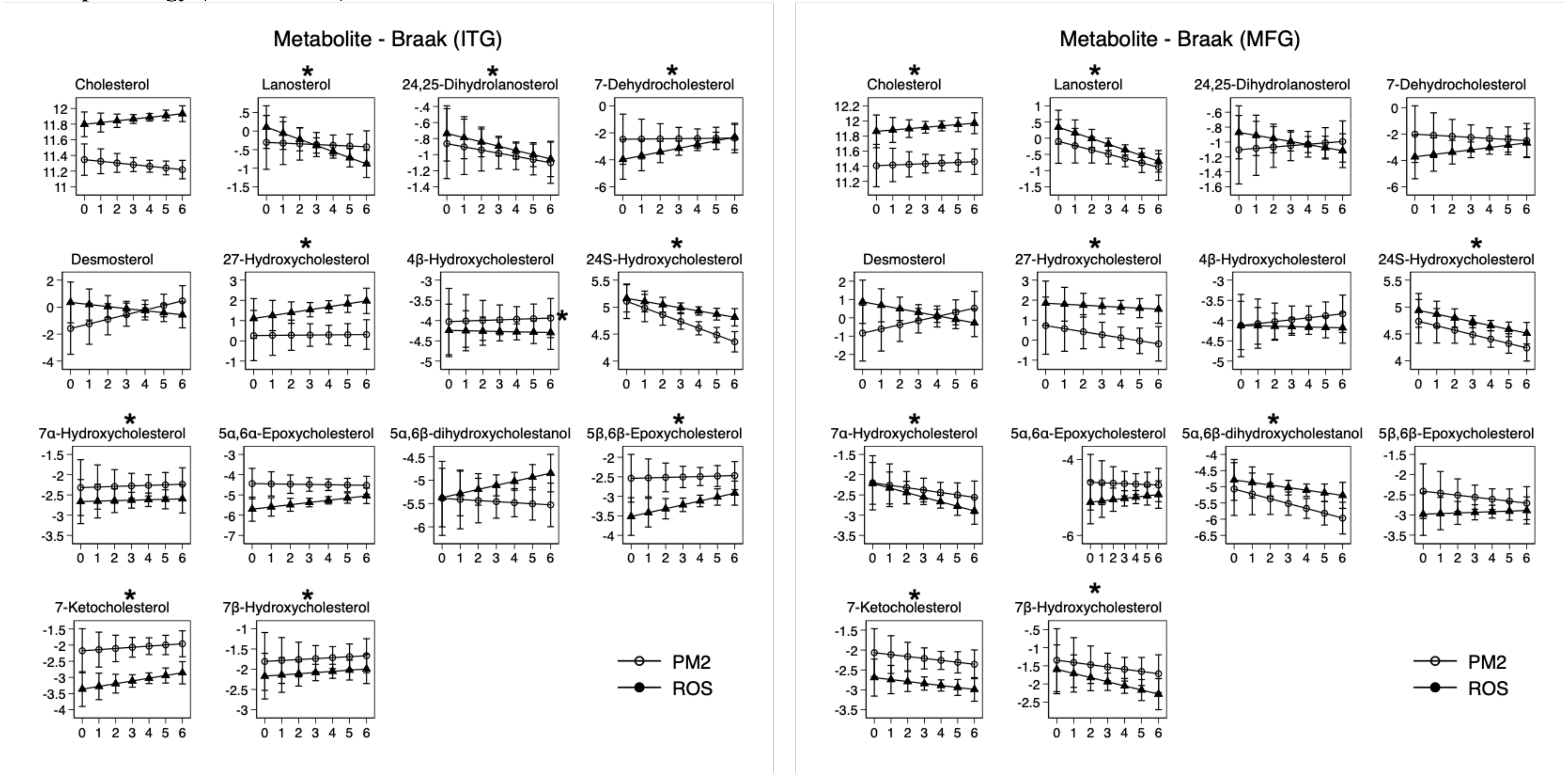

Alzheimer's disease (AD); Cognitively normal (CN); Asymptomatic AD (ASY); Inferior Temporal Gyrus (ITG); Middle Frontal Gyrus (MFG)

Convergent associations – where linear associations between metabolite concentration and disease status/ pathology in ROS and BLSA were in a similar direction – were pooled and indicated with a "*". For primary analyses (Table 1), linear mixed effects models were used for each of three a-priori-defined biochemical pathways (i.e. clusters): *de novo* cholesterol biosynthesis, cholesterol catabolism (enzymatic) and cholesterol catabolism (non-enzymatic). At least two metabolites indicated with a * within each cluster were required in order to run models; clusters where only one metabolite could be pooled are presented within cohort (i.e. study) specific secondary analyses (Supplementary Table

1). Error bars indicate 95% confidence intervals. Baltimore Longitudinal Study on Aging (BLSA) sample size: AD (n=15), CN (n=8); Religious Orders Study (ROS) sample size: AD (n=31), CN (n=22).
